# Theories Predicting End-User Acceptance of Telemedicine Use: Systematic Review

**DOI:** 10.2196/13117

**Published:** 2019-05-21

**Authors:** Lorenz Harst, Hendrikje Lantzsch, Madlen Scheibe

**Affiliations:** 1 Research Association Public Health, Center of Evidence-based Healthcare University Clinic Carl Gustav Carus Technische Universität Dresden Dresden Germany; 2 Master Program Health Sciences / Public Health Faculty of Medicine Carl Gustav Carus Technische Universität Dresden Dresden Germany; 3 Center for Evidence-Based Healthcare University Clinic Carl Gustav Carus Technische Universität Dresden Dresden Germany

**Keywords:** systematic review, telemedicine, technology, patient compliance

## Abstract

**Background:**

Only a few telemedicine applications have made their way into regular care. One reason is the lack of acceptance of telemedicine by potential end users.

**Objective:**

The aim of this systematic review was to identify theoretical predictors that influence the acceptance of telemedicine.

**Methods:**

An electronic search was conducted in PubMed and PsycINFO in June 2018 and supplemented by a hand search. Articles were identified using predefined inclusion and exclusion criteria. In total, two reviewers independently assessed the title, abstract, and full-text screening and then individually performed a quality assessment of all included studies.

**Results:**

Out of 5917 potentially relevant titles (duplicates excluded), 24 studies were included. The Axis Tool for quality assessment of cross-sectional studies revealed a high risk of bias for all studies except for one study. The most commonly used models were the Technology Acceptance Model (n=11) and the Unified Theory of Acceptance and Use of Technology (n=9). The main significant predictors of acceptance were perceived usefulness (n=11), social influences (n=6), and attitude (n=6). The results show a superiority of technology acceptance versus original behavioral models.

**Conclusions:**

The main finding of this review is the applicability of technology acceptance models and theories on telemedicine adoption. Characteristics of the technology, such as its usefulness, as well as attributes of the individual, such as his or her need for social support, inform end-user acceptance. Therefore, in the future, requirements of the target group and the group’s social environment should already be taken into account when planning telemedicine applications. The results support the importance of theory-guided user-centered design approaches to telemedicine development.

## Introduction

### Definition and Delimitation of Telemedicine and Related Terms

Telemedicine, as well as every other digital health technology, comes with the promise of changing care delivery for the better, be it by reaching traditionally underserved regions [1] or populations [2] or by enhancing patient-provider communication to facilitate shared decision making [3].

Digital health care is known under various terms, for example, telemedicine, electronic health (eHealth), telehealth, or, as new digital devices came to be used, mobile health (mHealth). This study focused on telemedicine, as telemedicine involves health care services being delivered by health care providers in a patient-centered manner, from a geographical distance, using ICT (Information and Communication Technology) [4]. The term is hereby clearly delimitated from other modes of digital care delivery. eHealth, for example, also encompasses electronic management of patient data, whereas telehealth covers the use of Internet of Things to enable self-management of health and the quantified self [5].

### Acceptance and Diffusion of Telemedicine

The possibly high potential of telemedicine can only be fulfilled if telemedicine reaches a high diffusion throughout the health care system. Nevertheless, although being used for over 50 years now [6], telemedicine still does not mostly overcome the pilot project stage [7] and therefore never prevails in regular care [8,9]. Among the most commonly used applications are those enabling digital data storage and exchange or telecounseling for diagnostic purposes [10,11]. Those, however, are not covered by the definition of telemedicine, as care is not directly delivered to the patient. Therefore, patient acceptance is not a relevant factor for those applications.

Rogers argues that innovations that are compatible with their environment are more easily adopted than those that are not suitable to past experiences of the adoption units [12]. However, telemedicine entails changes in patient-provider communication, patient assessment, and engagement [13,14]. As Riley et al argue, many of the applications deploy so-called *cues to action*, demanding behavior change by the end user [15].

Therefore, it is necessary to study the factors influencing end-user acceptance of telemedicine, even more so as acceptance is a prerequisite for adoption of an innovation and therefore its diffusion [12]. Nevertheless, acceptance is often reduced to the study of the usability, that is, certain design features of technology [16]. For the implementation of Health Information Systems, Ifinedo finds factors influencing Canadian nurses’ acceptance to go way beyond technical features [17]. Therefore, Hastall et al call for a holistic assessment of acceptance, incorporating not only features of the technology but also characteristics of the end user [18]. When structuring this holistic approach by studying individual, social, environmental, and technological factors of acceptance they recur on behavioral models of acceptance that have been proven to be effective in predicting behavior change in all sorts of health interventions—for example, by Sahay et al [19].

### Theoretical Background of Technology Acceptance

The oldest yet still widely used model for health behavior change is the *Health Belief Model (HBM)* that focuses on the individual assessment of vulnerability, outcomes and costs of behavior change, and external cues toward behavior change [20]. It can be applied, in combination with other theories, to explain user’s acceptance of wearables [21], which suggests transferability to telemedicine.

On the basis of the focus of the HBM on individual perceptions, the *Theory of Reasoned Action (TRA)* was formulated, also focusing on the attitude toward the intended outcome behavior but adding measures of subjective norm, that is, the perception of the behavior in question by those whose opinion is valued by the individual [22]. TRA is applicable for predicting attitude toward the use of a teleconsultation system in neurology [23]. Perceived control over one’s own health was added to the theory later on, developing the *Theory of Planned Behavior (TPB)* [24]. The TPB is applicable to the use of fitness apps [25]. TRA and TPB constructs were used by Davis to explain acceptance of technology as a precondition for its use. *The Technology Acceptance Model (TAM)*, along with several additions, defines use as predicted by attitude toward the use, which again is a function of perceived usefulness and perceived ease of use, both being value judgements of the design features [26]. The TAM was further developed by Venkatesh, adding several components from previous behavioral theories, such as performance and outcome expectancy from the Social Cognitive Theory [27] and subjective norm from TRA and TPB [28]. His final model is called the *Unified Theory of Acceptance and Use of Technology (UTAUT)*. As relevant predictors of acceptance vary between these 2 models, so do the definitions of technology acceptance within the models. Although in the TAM, acceptance is defined as “actual system use” [26], in the UTAUT, it is defined as “use behavior” [28]. Nevertheless, both models aim to study acceptance. Both TAM and UTAUT have been used excessively to explain the use of several digital health applications, such as data sharing systems [29] and assessment tools for cognitive functions [30].

In contrast to the aforementioned theories, where acceptance or use is a dependent variable, Normalization Process Theory postulates that collective action—that is, for the purpose of this paper, technology use—is one of several highly interconnected variables. Among them are group processes and organizing structures [31].

In addition to the predictors derived from the theories and models, other variables may have the potential to determine individual peoples’ willingness to use telemedicine. The nonadoption, abandonment, scale-up, spread, and sustainability (NASSS) framework lists several possible domains challenging the implementation process of telemedicine. The individual end user, on whom this study focuses, according to the NASSS framework, interacts with the organizational and societal context and is constrained by his or her medical status, as well as technological features [32]. The authors of the NASSS framework conclude that there is a lack of theoretical foundation for individual adoption processes. Such, the UTAUT is especially suitable to close this research gap, as it also encompasses the organizational and technological infrastructure in which the individual acceptance unit lives [28].

There is scarce evidence on which theory or model of technology acceptance or health behavior change is best suited to explain the acceptance and therefore use of telemedicine, as defined above [33,34]. Lai gives a comprehensive review on existing technology acceptance models and theories [35]. Evidence synthesis of theoretical predictors can be found solely for the acceptance of health information systems, such as eHealth records [36], yet it is not found for telemedicine defined as narrow as it is defined by Sood et al [4].

Individual studies that focus on theoretical components as predictors of the acceptance of telemedicine exist. However, there is still no systematic overview of theoretical components that are able to empirically explain the acceptance of telemedicine. This study aimed to fill this void by answering the following research question:

Which theoretical components are empirically associated with end-user acceptance of telemedicine?

## Methods

### Design

This systematic review was conducted according to the standardized strategy provided by the Cochrane Collaboration [37], and it also follows the *Preferred Reporting Items for Systematic Reviews and Meta*-*Analyses* (PRISMA) checklist [38]. A review protocol was created a priori and published at Prospero (Number CRD42018098658).

### Inclusion and Exclusion Criteria

The Population, Intervention, Comparison, Outcome, and Study Design (PICOS) criteria were used for deriving the inclusion and exclusion criteria for the review [37]. As this review aimed to explain the acceptance of telemedicine, and not the effectiveness, the comparison was omitted. The population studied included patients and health care providers as well as their respective direct social environment. As the term *telemedicine* is not consistently used, it cannot be clearly delimitated from related terms. Therefore, terms such as eHealth and mHealth were also part of the search strategy. Studies examining telemedicine that was used to deliver health care services in a patient-centered manner over a geographical distance were included. If this was not the case, those studies were excluded during the full-text assessment at the latest. Only studies aiming to explain end-user acceptance as a primary outcome using a theoretical underpinning were included. As the explanatory power of theoretical components was to be identified, only quantitative research designs were included. The same is true for studies published in English and German and for studies published in peer-reviewed journals. A further specification of the inclusion and exclusion criteria can be found in Table 1.

### Literature Search

The search string was a combination of the building blocks of the PICOS. Electronic searches were conducted in PubMed, as it is the most important and conclusive database for medical research, and PsycINFO in June 2018. PsycINFO was chosen as it is a database for psychological research, and it was therefore deemed likely to list studies featuring theoretical foundations for technology acceptance. For PubMed, Medical Subject Headings terms were used, whereas for PsycINFO, the functional equivalent, that is, the Thesaurus, was used. The term telemedicine is used ambiguously and partly synonymously with others [5]. As there are applications that are not explicitly called telemedicine but meet the telemedicine definition [39], different digital health terms, such as eHealth, mHealth, and telehealth, were included in the search string. The database-specific search strings can be found in the appendix (see Multimedia Appendix 1).

A hand search was conducted. In addition to a forward search in Web of Science, major publications in the field of telemedicine were searched, such as *Telemedicine and eHealth* and the *Journal of Medical Internet Research*. In addition, a search took place in the Institute of Electrical and Electronics Engineers Xplore database, which appears not to be designed for comprehensive search strings. To cover the research fields of informatics and information systems, a hand search was also conducted in the journals *Management Information Systems Quarterly* and the *American Journal of Information Systems*. References of the included studies were assessed to identify landmark studies, that is, those cited by more than one of the included papers.

**Table 1:** Inclusion and exclusion criteria for the review according to the Population, Intervention, Outcome, and Study Design scheme.

Category	Inclusion criteria	Exclusion criteria
Population	Patients, social environment (relatives and peers or peer groups), and health care providers	Nonhuman populations, not patients, not health care providers, and veterinarians
Intervention	Telemedicine-delivered patient-centered health care services with involvement of health care providers	No telemedicine, that is, no patient-centered health care services delivered, no involvement of health care providers
Outcome	Acceptance of health technologies on the basis of theoretical components	No theory-based factors (derived from correlations, causal models, eg, multivariate regression analyses or Structural Equation Modeling or effect strengths calculated from group comparisons), no statements about acceptance, and theories
Study design	Intervention studies (randomized or nonrandomized controlled trials), observational studies (cohort studies, cross-sectional studies, and case-control studies), and studies published in English or German language	Qualitative studies (in-depth interviews, expert interviews, focus groups, and delphi), reviews, editorials, letters to the editor, studies not published in English or German, or not published in peer-reviewed journals

### Identification and Selection of Studies

Predefined inclusion criteria were applied independently by 2 raters (LH and HL) to screen for potentially relevant titles and abstracts within all studies obtained from the database and the hand search. In a next step, all possibly eligible studies were subsequently screened as full texts, also by 2 independent reviewers (LH and HL). Articles that did not meet the aforementioned inclusion criteria were excluded. Each of the 2 reviewers documented the reasons for exclusion so that a direct comparison was possible and a transparent procedure was ensured. Any disagreement over the suitability of certain studies was discussed among the raters and resolved by consensus.

### Data Extraction and Presentation

Study characteristics (bibliographical information, study design, study population, type of telemedicine application, theoretical model or theory, and statistical methods) were extracted independently by the 2 reviewers (LH and HL). *Statistical methods* encompassed the dependent variable, significant predictors (rooted within theory), and measures for internal consistency and reliability as reported by the authors of the included studies, as well as the statistical analysis conducted. Disagreements were discussed, and a consensus between both extractors was reached. The entire extraction table was discussed by all authors (LH, HL, and MS). Only those predictors rooted within acceptance theories or models were extracted, which eliminated those added by the authors of the included studies to increase the variance explained. Both restrictions are in line with the research question.

For all theories discovered, as well as all the significant predictors, first frequencies and then variances explained and effect strengths were presented in tables. Afterward, median variance that was explained by each study, and median effect strengths of the theoretical predictors used within each study were also calculated and presented in tables. The median is a proper measure, as it is “the middle score of a set of ordered observations” [40]. Therefore, all statistical values for variance explained (R²) and each predictor (odds ratios [ORs] and betas) were listed and the middle value was either discernible (in an uneven list of values) or calculated as the arithmetic mean of the 2 middle values (in an even list of values) [40].

### Assessment of Methodological Quality

Quality assessment was conducted using the Appraisal Tool for Cross-Sectional Studies (AXIS Tool) for quantitative studies [41]. Quality assessment was conducted by 2 authors (LH and HL) independently. The focus of the assessment procedure was on methodological issues, such as the selection of study participants, the reliability and validity of the outcome measurements, and the consideration of potential confounding factors and bias in the results. On the basis of these criteria, an evaluation was carried out on a 2-step scale from 0=not satisfyingly explained to 1=satisfyingly explained. If items concerning methods and results (as explained above) were all rated 1, the risk of bias within the results of the study was deemed *low*, otherwise, it was deemed *high*.

## Results

### Search Results

The electronic database search resulted in 6188 potentially eligible articles. A hand search resulted in a total of 13 additional studies. After removing 283 duplicates, 5821 articles were excluded by independent screening of titles and abstracts. Of the resulting 97 full texts, 73 were excluded, as they did not meet the inclusion criteria. The main reason for study exclusion was the use of an inadequate intervention, that is, the intervention studied did not fall within the definition of telemedicine (36 times). For studies where the full text was not available, the authors were contacted. Owing to nonresponse, 7 full texts could not be procured until the end of August 2018.

**Figure 1:**
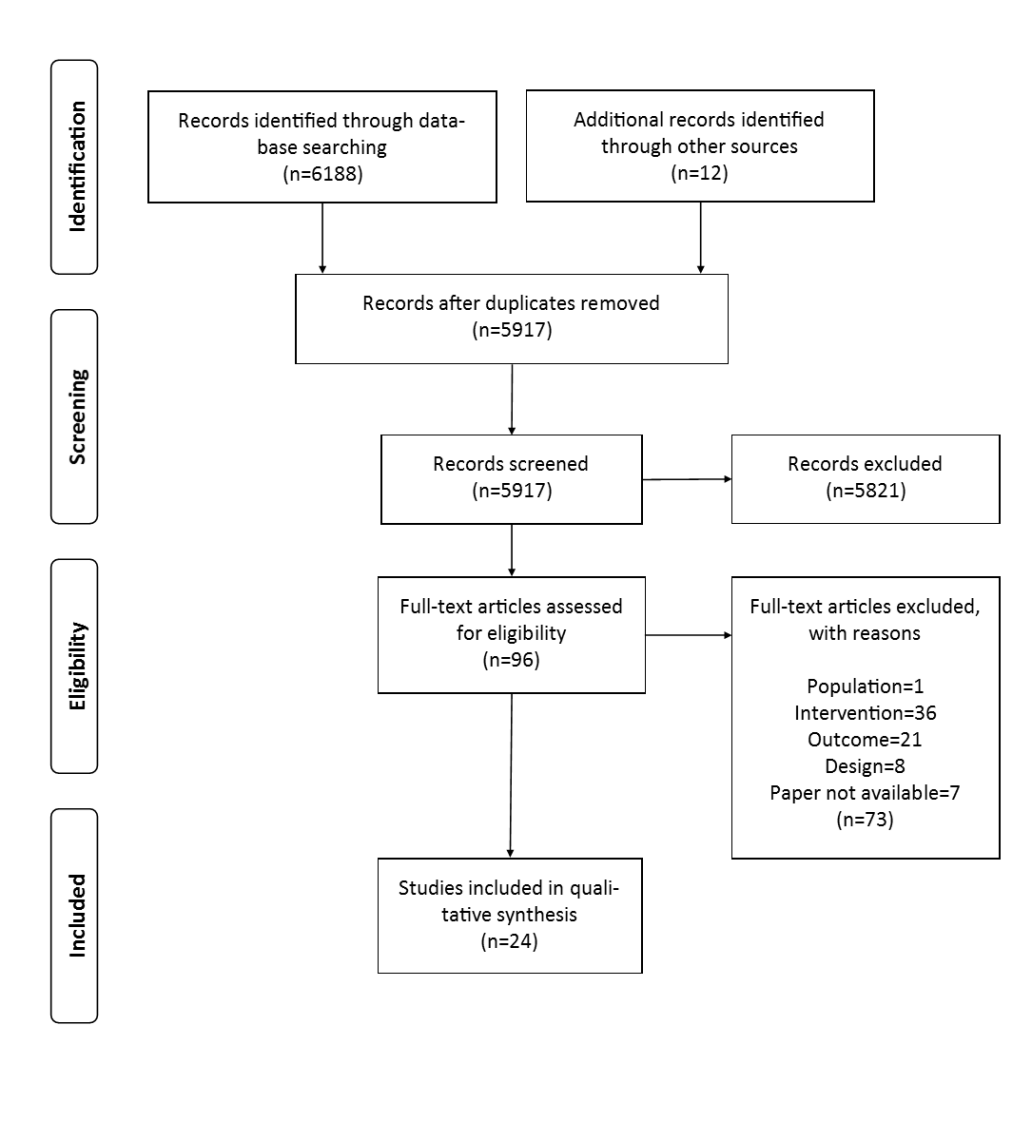
Flow chart of studies included and excluded from the systematic review.

Finally, 24 papers met the predefined inclusion criteria and formed the basis for data extraction. A total of 20 of the papers resulted from the application of the search strings in PubMed and PsycINFO, whereas 4 additional ones were uncovered by hand search. A total of 3 of the 4 were found in relevant journals in the field of telemedicine and information systems research and by checking references of the included studies for landmark studies, the third relevant study was included. The PRISMA flow chart in Figure 1 shows the process of the study selection.

A list of studies excluded during full-text screening, complete with the reasons for exclusion, can be found in the appendix (see Multimedia Appendix 2). Table 2 shows the most important data extracted for each study. For a list of all extracted data, please see Multimedia Appendix 3.

**Table 2:** Characteristics and outcomes of all included studies.

Author (year); Journal		Theoretical model or theory	Components of the model or theory with significant explanatory power	Effect strength and significance^a^
**Health care provider**				
	Asua et al (2012); BMC Medical Informatics and Decision Making	TAM^b^; DOI^c^; TIB^d^	PU^e^ (TAM); PEOU^f^ (TAM); *Extended TAM:* Compatibility (DOI); Facilitators (TIB)	*Original Technology Acceptance Model:* PU: OR^g^ 5.28^h^ (95% CI 3.14 to 10.01); PEOU: OR 1.93^i^ (95% CI 1.11 to 2.37); Final Model: Nagelkerke R²=0.63; *Extended TAM:* PU: OR 2.65^i^ (95% CI 1.15 to 6.12); Compatibility: OR 3.06^j^ (95% CI 1.30 to 7.18); Facilitators: OR 4.90^h^ (95% CI 2.38 to 10.09); Final model: Nagelkerke R²=0.72
	Gagnon et al (2012); Telemedicine and e-health	TAM	PU (TAM); *Modified model:* Facilitators (TIB)	*Original Technology Acceptance Model*:; PU: OR 5.28^h^ (95% CI 2.12 to 13.11); Nagelkerke R²=0.42; *Modified model:* Facilitators: OR 4.96^j^ (95% CI 1.59 to 15.55); Final model: Nagelkerke R²=0.54
	Hennemann et al (2017); Journal of Health Communication	UTAUT^k^	SI^l^; PE^m^	SI: beta=.37^h^ (95% CI 0.25 to 0.61); PE: beta=.28^h^ (95% CI 0.12 to 0.44); Final model: R²=0.63
	James et al (2016); Journal of Diabetes Science and Technology	TAM	PEOU; SN^n^	Independent Predictors of Diabetes Educators’ Intentions to Use; *Apps*: PEOU: OR 1.15^i^ (95% CI 1.07 to 1.31); Final model: R²=0.71; *Video conferencing*:; PEOU: OR 1.21^h^ (95% CI 1.08 to 1.35); SN: OR 1.21^j^ (95% CI 1.07 to 1.37); Final model: R²=0.68
	Kuhn et al (2015); Professional Psychology: Research and Practice	DOI	Complexity	Complexity: OR .35^h^ (95% CI 0.23 to 0.55); Final model: Nagelkerke R²=0.53
	Orruño et al (2011); Journal of Telemedicine and Telecare	TAM; TIB; TRA^o^	PU (TAM); PEOU (TAM); Facilitators (TIB)	*Original Technology Acceptance Model*; PU on intention: OR 8.4,^h^ (95% CI 3.4 to 21.0); PEOU on intention: OR 7.4,^h^ (95% CI 2.9 to 19.0); Nagelkerke R²=0.71; *Modified Technology Acceptance Mode:* Facilitators on intention: OR 9.9^h^ (95% CI 2.80 to 34.94); Final model: Nagelkerke R²=0.78
	Saigi-Rubió et al (2014); Implementation Science	TAM; DOI; TRA; TPB; TR^p^	Level of ICT use (TR); Optimism (TR)	Technology Readiness Index: Level of ICT Use (Spain): b=2.661^j^; Level of ICT Use (Columbia): b=1.212^j^; Optimism (Bolivia): b=0.484^h^; Final model: Nagelkerke R² (Spain): 0.275; Nagelkerke R² (Columbia): 0.161; Nagelkerke R² (Bolivia): 0.197
	Saigi-Rubió et al (2016); International Journal of Technology Assessment in Health Care	TAM; TPB; TRA	PU (cost reduction, quality of care; TAM); ATT^q^ (confidentiality and security; TAM); SN^r^ (patients, medical staff; TRA)	PU (cost reduction) on BI^s^: b=1.342^i^; ATT (security and confidentiality) on BI: b=0.798^i^; SN (patients) on BI: b=.583^j^; SN (medical staff) on BI: b=1.005^j^; Moderations: SN (patients)xPU (quality of care) on BI: b=.347^j^; SN (patients)xPU (cost reduction) on BI: b=.462^i^; SN (medical stuff)xPU (quality of care) on BI: b=.366^i^; SN (medical stuff)xPU (cost reduction) on BI: beta=.488^i^; SN (administration)xPU (cost reduction) on BI: beta=.571^i^; Final model: Nagelkerke R²=0.481; CI NS^t^
	Spaulding et al (2005); Journal of Telemedicine and Telecare	DOI	Relative advantage (provider); Relative advantage (patient); Observability; Trialability; Opinion leader present	Relative advantage (provider): r=0.42^i^; Relative advantage (patient): r=0.42^i^; Observability: r=0.57^i^; Trialability: r=0.44^i^; Opinion leader present: r=0.52^i^; CI NS
	van Houwelingen et al (2015); Journal of Gerontological Nursing	UTAUT	PU; EE^u^; SI	PU: beta=.435^h^; EE: beta=.28^h^; SI: beta=.216^i^; Final model: R²=0.54; CI NS
	Vanneste et al (2013); BMC Medical Informatics and Decision Making	UTAUT; SCT^v^	FC^w^ (UTAUT); SE (SCT)	FC: beta=.287^h^; SE: beta=.218^h^; Final model: R²=0.308; CI NS
	Zhang et al (2010); Computers, Informatics, Nursing	TAM 2	SN; IM^x^; PEOU; PU	SN: beta=.323^j^; IM: beta=.227^j^; PEOU: beta=.35^j^; PU: beta=.422^h^; R²=0.375; CI NS
**Patients**				
	Cajita et al (2017); Journal of Cardiovascular Nursing	TAM; TIB	PEOU (TAM); PU (TAM)	*Block 5:* change in R²=0.095^h^; PEOU: beta=.16^h^ (95% CI 0.07 to 0.24); *Block 6:* change in R²=0.130^h^; PU: beta=.33^h^ (95% CI 0.24 to 0.41), Final model: R²=0.353
	de Veer et al (2015); BMC Health Services Research	UTAUT	PE; EE; SE	*PE:* Block 2: beta=.52^h^; Block 3: beta=.24^h^; Block 4: beta=.24^h^; Final model: beta=.24^h^; *EE:* Block 3: beta=.42^h^; Block 4: beta=.42^h^; Final model: beta=.35^h^; *SE:* Final model beta=.01^j^; Final model: R²=0.41; CI NS
	Dockweiler et al (2017); Gesundheitswesen	UTAUT	PE; EE	*PE* (5 significant variables); average effect: OR 11.325^i^ (95% CI 2.666 to 49.015); *EE* (2 significant variables); average effect: OR 0.121^i^ (95% CI 0.022 to 0.685); Final model: R²= 0.765
	Dou et al (2017); JMIR Mhealth Uhealth	TAM; TAM 2; Dual-Factor model; HBM^y^	PU (TAM); PHT^z^ (HBM); Resistance to change (Dual-Factor Model)	PU on intention to use: beta=.616^j^; PHT on intention to use: beta=.305^j^; resistance to change on intention to use: beta=−.149^i^; Final model: R²=0.412; CI NS
	Hennemann et al (2016); Journal of Medical Internet Research	UTAUT	SI; PE; EE	SI: beta=.39^j^ (95% CI 0.3 to 0.54); PE: beta=.31^h^ (95% CI 0.19 to 0.43); EE: beta=.22^h^ (95% CI 0.09 to 0.31); Final model: R²=0.78
	Hossain et al (2018); Telemedicine and e-Health	UTAUT; TAM	Social reference (means SI; UTAUT); ATT (TAM); FC (UTAUT)	SR: OR 9.73^j^ (95% CI 4.16 to 22.78); ATT: OR 4.56^j^ (95% CI 2.71 to 7.66); FC: OR 3.92^i^ (95% CI 1.29 to 11.95); Final model: R²=0.55
	Huygens et al (2015); Interactive Journal of Medical Research	UTAUT	EE; PE; FC; SI; ATT	Service to ask questions by internet via email or a website: EE: OR 5.46 (95% CI 3.27 to 9.13); PE: OR 5.47 (95% CI 3.44 to 8.70); ATT: OR 5.85 (95% CI 3.63 to 9.43); FC: OR 7.91 (95% CI 4.53 to 13.82); SI: OR 4.34 (95% CI 2.46 to 7.68); No levels of significance reported
	Lin and Yang (2009); Telemedicine and e-Health	TAM	PU; ATT; SN; PEOUxATT; PUxATT; SNxATT	*Direct effects:* ATTon BI (lambda=.76^j^); SN on BI (lambda=.16^i^); *Total effects*; ATT on BI (lambda=.76^j^); PU on BI (lambda=.62^j^); SN on BI (lambda=.42^j^); PEOU on BI (lambda=.3^j^); Final Model: R²=0.8; CI NS
	Peeters et al (2012); Journal of Clinical Nursing	DOI	Relative advantage; Compatibility; Complexity; Observability	Relative advantage: beta=.17^i^; Compatibility: beta=.2^j^; Complexity: beta=.19^j^; Observability: beta=.34^h^; Final model: R²=0.61; CI NS
	Rho et al (2015); Cluster Computing	UTAUT	PE; EE; SI; FC on EE; FC on PE	PE: beta=.345^j^; EE: beta=.227^i^; SI: beta=.246^i^; FCxPE on BI: beta=.176^j^; FCxEE on BI : beta=.153^j^; Final model: R²=0.44; CI NS
	Zhang et al (2017); Informatics for Health and Social Care	TAM; SCT; PMT	PU (TAM); PEOU (TAM)*PU; SE (Protection Motivation Theory)*PEOU*PU* AI; RE (Response Efficacy)*PEOU*PU*AI; RE (Protection Motivation Theory)*PEOU*PU* AI	*Direct effect:*; PU: beta=.3^h^; *Moderator:*; PUxSE: beta=.145^j^; PUxRE: beta=.359^h^; Final model: R²=0.501; CI NS
**Social environment**				
	Jen and Hung (2010); Telemedicine and e-Health	TPB; TAM	ATT; PU; PEOU	BI of adopting Mobile Health Services is explained directly by ATT; ATT on BI (beta=.547^j^); Final model: R²=0.641 of the variance in BI; CI NS

^a^Italics serve as subheadings for stepwise models.

^b^TAM: Technology Acceptance Model.

^c^DOI: Diffusion of Innovations Theory.

^d^TIB: Theory of Interpersonal Behavior.

^e^PU: Perceived Usefulness.

^f^PEOU: Perceived Ease of Use.

^g^OR: odds ratio.

^h^*P* ≤.001.

^i^*P* ≤.05.

^j^*P* ≤.01.

^k^UTAUT: Unified Theory of Acceptance and Use of Technology.

^l^SI: Social Influence.

^m^PE: Performance Expectancy.

^n^SN: Social Norm.

^o^TRA: Theory of Reasoned Action.

^p^TR: Technology Readiness.

^q^ATT: attitude.

^r^SN: Social Norm.

^s^BI: Behavioral Intention.

^t^NS: not specified.

^u^EE: Effort Expectancy.

^v^SCT: Social Cognitive Theory.

^w^FC: Facilitating Conditions.

^x^IM: Image.

^y^HBM: Health Belief Model.

^z^PHT: perceived health threat.

### Study Characteristics

The publishing years ranged from 2005 to 2017. Among the included studies, the Netherlands (4 times) and Spain (4 times) stand out. Only 1 study comes from a developing country, that is, Bangladesh. The number of journals from different research fields shows the conclusiveness of the conducted search.

All studies were cross-sectional studies. The number of participants ranged from 84 to 1014 (mean n=266.25 (SD 210.07), median n=228). Accordingly, acceptance was tested for using inferential statistics in all cases. Only 1 study did not apply causal statistics but merely a correlation analysis [42]. Of the remaining 22, 7 conducted complex models of causality, Structural Equation Modeling (SEM) or Path Modeling. For the statistical analysis conducted within each study, see Multimedia Appendix 3.

Only 1 study relied completely on an existing, previously tested questionnaire for the applied theory or model [29]. All other studies applied self-developed questionnaires. However, a total of 3 studies did not specify how their questionnaire was tested for validity and reliability [42-44]. Of the remaining 20, 6 [29,45-49]—those using SEM—applied confirmatory factor analysis, whereas the others relied on Cronbach alpha statistics [29,45,47-62].

### Participant Characteristics

The population is balanced regarding health care providers and patients, as both were studied 11 times. Only 1 study focused on the social environment, namely relatives, as the key population.

Only 8 of the included studies reported a mean age, ranging from 43.53 to 77.8 years. This led to an overall mean age of 53.72 (n=8) years. The overall mean age for patients was 61.93 years, whereas the overall mean age for health care providers was 47.1 years. The remaining 15 studies reported age groups with different cohorts, which makes it impossible to include them into calculations of an overall mean age.

The overall percentage of female participants was higher among the 10 studies focusing on health care providers (1 did not report on gender) than among the 11 studying patients’ acceptance (77.3 vs 41.4).

### Telemedicine Applications

A wide variety of telemedicine applications were analyzed within the included studies. First, it should be noted that of the 24 studies, in 8 cases, the authors did not specify the type of telemedicine application they were studying, that is, they used generic terms such as eHealth [50-52] or telemedicine [42,43,45]. A total of 6 studies focused on mobile apps [46-48,53,54,63], whereas 3 were concerned with applications based on internet devices, such as Web-based aftercare [29,44,55]. A total of 2 applications were used for monitoring of disease parameters [56,57]. A total of 4 more applications targeted certain diseases [49,58-60].

It is also noteworthy that even if a certain telemedicine application was focused on, sometimes the application did not (yet) exist. Instead, certain features were shown to the participants, who were then asked to imagine whether they would be willing to use the would-be application [50,60].

Although it was intended beforehand to study acceptance relative to broader categories of telemedicine applications [39], the sometimes quite generic terms used in the included studies do not allow for such a nuanced analysis. For an overview of telemedicine applications studied, see Multimedia Appendix 3.

### Medical Conditions

Along with the concrete telemedicine application, a target disease or medical condition for which telemedicine was supposed to be used was also not stated in 8 of the included studies [42-44,50-52,57,61]. A total of 8 studies dealt with the acceptance of telemedicine applications for chronic diseases, such as diabetes [45,58]. Furthermore, in 3 cases, mental health conditions were studied [54,55,60]. Among the remaining medical conditions targeted were skin lesions [59] and heart failure [53]. For an overview of medical conditions studied, see Multimedia Appendix 3.

### Relevant Models and Predictors

#### Frequency of and Variance Explained by Theories and Models

The results are presented as follows: First, frequencies of the acceptance theories and models used within the 24 included studies are reported, along with their median variance explained, as calculated by the authors. Calculations were thought to be justified, as the theories and models studied proved applicable across a wide variety of telemedicine and medical conditions. Afterward, frequencies and median effect strengths of the predictors found to be significant by the included studies are presented. Throughout the Results section, a distinction regarding relevance of the theories and predictors will be made according to health care providers, patients, and their social environment (see Table 2).

As depicted in Table 3, the TAM is used 11 times within the included studies of this review, and therefore most often. The UTAUT, however, is used 9 times. It should be mentioned that the UTAUT was used 7 times, without any additional predictors from other models, whereas the same is true for the TAM only in 4 cases. Instead, it is used with a variety of other models, among them are the TRA [43,59] and the TPB [46]. The so-called TAM 2, an extension of the original TAM, [64] was used 2 times within the included studies.

Contradictory to that, the TAM still has the highest amount of variance explained among all the models included in this review: A median R² of 0.68 is achieved by the TAM, compared with an R² of 0.59 for the UTAUT. The TAM was used more often than the UTAUT, whether in combination or not.

The Diffusion of Innovations Theory by Rogers was used to explain acceptance far less (3 times), yet it still reaches a median R² of 0.57, which is, in part, because of the fact it was once used in combination with the TAM [56].

There was no other theory or model used alone, except for the TAM or the UTAUT, the Theory of Interpersonal Behavior (TIB) being one of the remaining, which was used in combination with others most often—3 times [53,56,59].

The most powerful combination of models is based on the TAM, adding components of the TIB and the TRA, with a variance explained of R²=0.78 [59]. This R², however, is still lower than the one achieved by Lin and Yang, using only the TAM, which was 0.8 [49]. The only significant predictor added by the TIB, according to Orruño et al, is the presence of facilitators. The TRA does not add significant predictors at all.

The TAM was used to explain acceptance of health care providers 7 times and 3 times for the acceptance of patients. The median proportion of variance explained by the TAM was higher for health care providers (R²=0.63) than for patients (R²=0.501).

The UTAUT was used more often to explain acceptance of patients than of health care providers (5 vs 3 times). For patients, its median explanatory power is also higher (R²=0.55) than for health care providers (R²=0.54). For frequencies of the models and theories, as well as their median variance explained, see Tables 3 and 4. A complete list of combined models and theories can be found in the appendix (Multimedia Appendix 4).

**Table 3:** Frequency of theories and models used to explain acceptance.

Model/Theory	Frequency of use
Dual factor model	1
Health Belief Model	1
Protection Motivation Theory	1
Technology Readiness	1
Social Cognitive Theory	2
Technology Acceptance Model 2	2
Theory of Interpersonal Behavior	2
Theory of Planned Behavior	2
Theory of Reasoned Action	2
Diffusion of Innovations Theory	3
Unified Theory of Acceptance and Use of Technology	9
Technology Acceptance Model	11

**Table 4:** Median variance explained by each model alone (if theory or model was used alone).

Model/Theory and variance explained (per author)		Median variance explained
**Technology Acceptance Model**		
	0.35 (Cajita et al)	0.68
	0.42 (Gagnon et al)	0.68
	0.63 (Asua et al)	0.68
	0.68 (James et al)	0.68
	0.71 (James et al)	0.68
	0.71 (Orruño et al)	0.68
	0.80 (Lin and Yang)	0.68
**Unified Theory of Acceptance and Use of Technology**		
	0.41 (de Veer et al)	0.59
	0.44 (Rho et al)	0.59
	0.54 (van Houwelingen et al)	0.59
	0.63 (Hennemann et al)	0.59
	0.77 (Dockweiler et al)	0.59
	0.78 (Hennemann et al)	0.59
**Diffusion of Innovations Theory**		
	0.53 (Kuhn et al)	0.57
	0.61 (Peeters et al)	0.57
**Technology Acceptance Model 2**		
	0.38 (Zhang et al)	0.38

#### Frequency and Effect Strength of the Significant Predictors

UTAUT adds, among others, the predictor *social influence* to the basic TAM predictors. As a result, it accounts for the perception of an item of technology by others, whose opinion is valued by the individual. The predictor was uncovered as a significant predictor by 6 of the included studies and comprises the attitudes of colleagues, patients, or the direct social environment, such as families and friends, toward telemedicine [55,58,60]. The predictors *performance expectancy*, *effort expectancy*, and *facilitating conditions* (sometime just called *facilitators*), all part of the UTAUT, were used 6 times as well.

The predictor mentioned as significant most often (11 times) was *perceived usefulness*, which is not part of the UTAUT but of the original TAM. It reaches both high ORs (when logistic regression was performed) and high betas (when multiple linear regression was performed). From a patient perspective, usefulness is achieved when, for example, telemedicine use improves quality of life or makes the care process more convenient for the patient [48]. For health care providers, according to the studies included, usefulness is mainly associated with streamlining care processes, such as diagnosis and monitoring of disease parameters [56,59]. The other TAM predictor, *perceived ease of use*, was discovered to be significant 6 times. It mostly covers the degree of training it would take, both patients and health care professionals, to understand and learn how to use the telemedicine application in question [55,56], and it is sometimes used synonymously with effort expectation [45].

*Attitude* was a significant predictor in 6 cases. However, in some of the proposed SEMs, it was circumvented in favor of direct effects of perceived usefulness [46] and perceived ease of use [49]. Yet, *attitude* is the predictor with the highest beta, with regard to the studies using multiple linear regression (median beta=.76).

When studying both median ORs and median betas, only the height of the numbers is reasonably interpretable, as calculation of both values differs greatly. Then, *Perceived usefulness* is the most important predictor for acceptance by health care providers, with a median OR of 5.28 and a median beta of .43 (as calculated by the authors).

Taking into account both OR and beta, there are 2 almost equally important predictors for patient acceptance of telemedicine: *performance expectancy*, with a median OR of 8.4 and a median beta of .3, and *social influence*, with a median OR of 7.04 and a median beta of .25. Patients expect telemedicine to help them cope with their health problems and thereby improve their health [45,55].

For relatives, *attitude* toward an mHealth care service used to connect their elderly family members with health care providers is the most important predictor (beta=.55). Lin and Yang operationalize attitude as the willingness to use a telemedicine application, as it is considered the ideal solution for a given health problem [49]. As this was the only study focusing on the social environment of patients, no further analysis was conducted.

The dependent variables used in the 24 studies do not always fit those intended by the authors of the original model. *Intention to use* telemedicine (or synonyms such as *behavioral intention*) was used 19 times, probably as only 2 studies reported actual use of their application [54,61], the actual dependent variable in the TAM. *Acceptance* and *adoption* were each used once. For a complete list of frequencies and effect strengths of all predictors, see Table 5. The following predictors are not listed as they were each mentioned only once in the included studies: Perceived Health Threat (beta=.305), Resistance to Change (beta=.149), Trialability (r=0.44), Opinion Leader present (r=0.52), Image (beta=.227), Optimism (beta=.484).

**Table 5:** List of predictors of acceptance according to frequencies of use, odds ratios, betas, b’s, and r’s.

Factors affecting telemedicine acceptance	*P* value, median	n	Odds ratio, median	Beta/lambda, median	b, median	r, median
Perceived usefulness	.001	11	5.28	.43	1.34	—^a^
Performance expectancy	.001	6	8.4	.3	—	—
Perceived ease of use	.01	6	1.57	.26	—	—
Effort expectancy	.001	6	2.79	.25	—	—
Facilitating conditions/faciliators	.001	6	4.96	.29	—	—
Social influence	.01	6	7.04	.25	—	—
Attitude to use	.01	6	5.21	.76	—	—
Subjective norms	.01	5	1.21	.16	.58	—
Relative advantage	.05	3	—	.17	—	0.42
Compatibility	.01	2	3.06	.2	—	—
Complexity	.006	2	0.35	.19	—	—
Self-efficacy	.051	2	—	.01	.22	—
Observability	.026	2	—	.34	—	0.57
Level of ICT use	.018	2	—	—	1.94	—

^a^No data provided.

### Methodological Quality Assessment

All but 1 study have a considerable risk for bias, as there is only 1 study in which all AXIS items from the Methods and Results section could be rated 1, which is the study on mHealth use intention of heart failure patients by Cajita et al [53]. It achieves 19 from a total of 20 points.

The objective of each study (24 times rated with 1), study design (24), sample size (24), as well as sample frame (22), was well described in most studies. Moreover, questions dealing with the measurement of outcome variables and the determination of statistical significance could always be rated 1.

Only a few studies have taken measures to address and categorize nonresponders. The evaluation of question 11—whether there was sufficient description of the study design and statistical methods applied to derive the results—shows that only about half of the studies have sufficiently described those, which is because of the fact that these studies have not reported any CIs or nonsignificant results. This diminishes the possibility to repeat their results. Moreover, half of the studies do not score high on questions 13 and 14, as their response rate is low, and there is no information provided about nonresponders, raising the question of nonresponse bias. Overall, the 24 included studies reached an AXIS score of 15.67. An overview of the rating for quality assessment according to the AXIS Tool can be found in the appendix (Multimedia Appendix 5).

## Discussion

### Importance of Acceptance Theories and Models in General

The results of the 24 original studies included in this systematic review support Hastall et al’s demands for a holistic analysis of technology acceptance in health care by relying on a theoretical background [18].

The UTAUT was used more often to explain acceptance for patients than for health care providers, which is likely because of the fact that the UTAUT includes variables of the construct, *social influence*. Those are also more important for patients than for providers. For health care providers, the TAM has the highest variance explained, relying also on the predictor *perceived usefulness*, which reaches both high ORs and betas.

It is noteworthy that, although it is still the most commonly used model, the TAM is combined with further predictors 7 times. Those predictors borrow heavily from the TIB, TPB, and TRA, thereby enabling the original TAM to also incorporate factors of acceptance not only rooted within technology but also rooted within the individual as the end user. The high prevalence of the UTAUT, used 9 times and only 2 times in combination, also adds to the importance of such factors, being a far more holistic model than the TAM. Together, these findings support Karsh’s statement that acceptance is not solely achieved by improving usability [16]. Apart from that, they fulfill, in parts, the demand articulated by Riley et al for development and validation of novel health behavior theories for mobile interventions. Such theories should, according to the authors, include features and attributes of the technology as well as characteristics of the end users [15].

### Differences Between Patients and Health Care Professionals

Among the predictors added to the TAM, which are already part of the UTAUT, the most prevalent are those covering the social and organizational environment of the individual, for example, *social influence* and *facilitating conditions*. The latter supports results from qualitative observational research on telehealth readiness by older *patients* done by van Houwelingen et al. The authors conclude the need for easily available sources for technological support in case of problems with technology use [65]. *Social influence*, when phrased positively, can be understood as *social support*, and it was uncovered to be an important factor in technology acceptance, for example, for Web-based interventions for pregnant women by Berg et al [66]. Venkatesh et al included *social influences* into the UTAUT to pay respect to the fact that individuals’ acceptance behavior is being influenced by what they assume others might think about them when using a certain technology [28]. The results presented here show this is also true for telemedicine. However, they contradict those presented by Boessen et al, who state that intrinsic motivation to use a self-management tool trumps the perception of others, which serves as extrinsic motivation [67]. However, Peeters et al show that for people living alone, the positive effects of telemedicine use are much more observable (as defined by Diffusion of Innovations Theory), and so they were also more willing to adopt home telecare than those living with a partner or relative [62]. Although these results seem contradictory to the importance of social influence, they give another meaning to the concept, showing that telemedicine can, when used properly, provide social contact where there is none.

Social influence appears to be more important for patients than for *health care providers*. For the latter, *perceived usefulness* is the most important predictor (studying median ORs and betas), which is in line with the results provided by Mothuy-Blanc et al. They show that whether psychotherapists are willing to use telepsychotherapy is predicted only by whether they find it useful [68]. As the provision of the best treatment to the patient is every health care provider’s primary concern, these results are not surprising. This can also explain why the TAM, focusing on perceived usefulness and perceived ease of use, is much more important for health care providers than for patients.

Performance expectancy is an important predictor for both patients’ and providers’ acceptance. This makes their individual expectations toward the outcome of telemedicine use an important focus for further research, even though it is contradictory to the results of Koivumäki et al. They found no significant influence of performance expectancy on the adoption of digital preventive services [69].

The data presented here show the importance of easy-to-use applications, as perceived ease of use was shown to be a significant predictor 6 times, as well as effort expectancy. Scheibe et al show that design features, such as simple, intuitive menus, large icons and high color contrasts, are especially important for older users [70]. As time is always scarce in health care provision, easy-to-use technology is also important for health care providers. De Angelis et al can even show that health care providers are willing to disseminate health information via Facebook, mainly as the Social Network is easy to use [71].

### Practicability of Technology Acceptance Models and Theories

On a more general level, the results presented here show that technology acceptance theories, as well as their basic behavioristic underpinnings, are applicable to the study of acceptance of telemedicine, even though they are quite old (the TAM was formulated in 1989, and the TRA was formulated in 1975). This is true despite the fact that none of those models and theories were originally formulated to fit health care technologies.

Moreover, the theories and models analyzed here are applicable to health care providers and patients alike, not to mention special types of diseases or telemedicine applications. There seems to be no special diagnosis that impacts acceptance and its preconditions, although it is noteworthy that there were not any studies focusing on telemedicine for patients with a cognitive impairment applicable for analysis. A pooling of data to calculate medians therefore seemed feasible.

Although an analysis of acceptance regarding different types of telemedicine applications was intended, only a superficial count of applications studied was feasible because of a lack of specification in some studies. However, no matter what kind of application was studied, the theories and models are applicable. If any insights can be gained from the few studies analyzing acceptance of a certain type of application, it is that acceptance is less of an issue when the basic device used is already familiar to the end user from everyday life, such as mobile phones or Web portals [72]. The results provided by Saigí-Rubió et al in their study from 2014 further stress this point, as they find previous use of ICT in their everyday life to be a significant predictor for physicians’ telemedicine use [73].

The applicability of the UTAUT, according to the variance explained, stresses the importance of holistic models of acceptance, incorporating not only characteristics of the individual adopter but also of his or her direct or indirect social environment. Facilitating conditions, being mentioned 6 times as relevant predictors, refer to the technological as well as the organizational infrastructure fostering acceptance. In a qualitative study by Cimperman et al, cost of the technology in use was mentioned as a major concern of older adults [74], which may appeal to funding agencies, such as insurance companies, to provide financial support.

It should be noted in this context that, although standardized and well-tested questionnaires exist for the TAM [26], as well as for the UTAUT [28] and the Diffusion of Innovations Theory [75], only 1 author cited here fully relied on the UTAUT questions suggested by Venkatesh et al in the original publication [29]. The remaining authors either made additions or changed the wording of several items. As none of the above mentioned models—nor the questionnaires used to test them—were originally developed for health care technologies, additions and changes to the questionnaires seem logical.

From a purely scientific standpoint, this research is proof that testing of theories and models in a variety of settings can be done by applying a systematic review of empirical studies, that is, by solely relying on secondary data.

### Limitations

This study has several limitations. First, papers not published in English or German were excluded, which may constitute a selection bias (language bias). Although a comprehensive search strategy has been used, and an additional hand search was conducted, it is possible that some relevant studies were missed if the specific keywords were used neither by the authors nor by the databases searched. Apart from that, a publication bias toward positive results cannot be precluded.

All included studies were cross-sectional studies. It should be noted that acceptance or the decision to adopt an innovation is a dynamic process, taking place over time [12], especially when health behavior change is going hand in hand with it [76]. Such processes cannot be captured with a cross-sectional study that, by nature, only covers 1 point in time [62].

Another limitation might be that acceptance cannot be fully evaluated regarding different medical conditions, as these were not often indicated in the included studies. The same is true for concrete applications. Acceptance is most likely rated differently when the subject is given the chance to use a real application instead of having to rate acceptance of hypothetical applications or generic terms such as eHealth or telemedicine.

Furthermore, age and gender could be confounders in the interpretation of the results, as they differ in the individual studies. However, because of questionnaires tailored specifically for each research interest in each included study, the theories, models, and predictors are applicable despite such heterogeneity in demographic variables.

In terms of quality assessment, the AXIS tool does not provide a numerical scale for assessing quality of the studies. Thus, a subjective rating, depending on the research interest, is required. Although the authors of the tool state that this subjectivity provides greater flexibility in assessing the quality of a study [41], this can still be a limitation.

Owing to the heterogeneity across the statistical methods within the included studies, a meta-analysis could not be performed. The medians, as well as the SDs calculated here, are an approximation, based on the overall applicability of the theories and models. It shows that the theories and models, as well as the predictors, can be compared.

The review strictly followed the PRISMA. We deployed a concise and literature-based search string, which was critically reviewed by several peers. The methodology applied for this review was checked for validity by the PROSPERO foundation, where a protocol was registered beforehand. A thorough quality assessment with a focus on methodological issues was conducted for each included study.

### Further Research Needs

As demonstrated in the review, social influence, also called social support, represents an important factor in telemedicine acceptance. The acceptance of telemedicine may benefit from the support of others perceived as important by the unit of adoption. In addition, telemedicine should be perceived as useful by the users or those who recommend them. All in all, models that are explicitly suited for health care technologies, such as telemedicine, need to be developed and empirically tested. The Health Information Technology Acceptance Model proposed by Kim et al in 2012 can serve as suitable model [77]. Even though it is based on existing technology acceptance and health behavioral theories, it was used in none of the studies found in this review.

This review highlights a lack of methodologically adequate studies. Future studies should have a longitudinal design and should consider the dimension of time, to allow for measuring the influence of mid- and long-term use of an innovation [78]. An important issue for further research in theory-based approaches to the measurement of acceptance is that the studies should examine acceptance of real telemedicine applications.

Complex, interdependent interactions within an organizational setting should also be tested for. The Normalization Process Theory can be helpful in this endeavor, as it proposes a nonlinear understanding of acceptance [31]. Theoretical factors promoting the implementation of telemedicine within a whole health care organization are not covered by the primary aim of this review. Yet, information about them can be derived, insofar as they can be subsumed under facilitating conditions. Little information was also found on predictors of telemedicine acceptance for relatives and peers of the primary users. Applying the NASSS framework, Greenhalgh et al find the complex interactions among the 6 levels, especially the organizations and the wider policy system, to be severely hindering telemedicine implementation [79]. The role of a supportive policy system as a predictor for telemedicine acceptance in a health care organization has been shown to be especially important in developing countries by Zailani et al. In addition, the authors stress the importance of the existing health culture as a mediator between individual technology assessment and telemedicine acceptance [80]. A more thorough investigation of the role played by the social environment of the end user, especially relatives, should also be conducted. Finally, the research presented here stops with the acceptance of telemedicine. However, another prerequisite of sustained use is task performance, that is, the ability to successfully use a technology [12]. Little research has been done on task performance in telemedicine use. However, Serrano and Karahanna have shown, on the basis of the task-individual-technology-fit theory, that skills in acquiring knowledge, problem-solving, and presenting solutions influence the successful use of teleconsulting systems [81]. Taking these results as a starting point, a shift from acceptance to performance research is feasible.

### Conclusions

The results of this systematic review indicate that acceptance of telemedicine can be examined by using technology acceptance theories and models. On the basis of the included studies, acceptance was most often predicted by perceived usefulness, social influences, and attitude. To examine how adoption processes evolve over time, longitudinal research on existing applications would be advisable in the future. A brief summary of the study results can be found in Textbox 1.

**Brief summary of the study results.**

What this study adds:

- Theories of technology acceptance are superior to common behavioral theories in explaining telemedicine acceptance.
- Not only features of the technology but also individual characteristics of the end user have to be considered when designing user-centered telemedicine.
- For patients, telemedicine acceptance of their social environment is crucial, as friends and families can support uptake of telemedicine use.
- For health care providers, usefulness of telemedicine in their clinical practice is of vital importance.

## Appendix

Multimedia Appendix 1 Database-specific search strings.

Multimedia Appendix 2 Excluded full texts with reasons for exclusion.

Multimedia Appendix 3 Complete list of data extracted from all 24 included studies.

Multimedia Appendix 4 List of models and theories used in combination.

Multimedia Appendix 5 Results of quality assessment according to the Appraisal Tool for Cross-Sectional Studies.

## Abbreviations

eHealth: electronic health
HBM: Health Belief Model
ICT: Information and Communication Technology
mHealth: mobile health
NASSS: nonadoption, abandonment, scale-up, spread, and sustainability
OR: odds ratio
PICOS: Population, Intervention, Comparison, Outcome, and Study Design
PRISMA: Preferred Reporting Items for Systematic Reviews and Meta-Analyses
SEM: Structural Equation Modeling
TAM: Technology Acceptance Model
TIB: Theory of Interpersonal Behavior
TPB: Theory of Planned Behavior
TRA: Theory of Reasoned Action
UTAUT: Unified Theory of Acceptance and Use of Technology
