# Supplementary material for: Theories Predicting End-User Acceptance of Telemedicine Use: Systematic Review
Source: J Med Internet Res. 2019 May 21;21(5):e13117. doi: 10.2196/13117 (PMC6547771; doi:10.2196/13117)
Supplement: Multimedia Appendix 1 [file jmir_v21i5e13117_app1.pdf]

Search String PubMed

Population	“health personnel” [Mesh] NOT “Veterinarians” [Mesh] OR patients [Mesh] OR patient OR patients OR relative* OR family OR families OR peer* OR “peer group” OR “peer groups“	8343887
AND		
Intervention	telemedicine* OR telemedicine [Mesh] OR telehealth OR Telematic* OR ehealth OR e-health OR E-Health OR “electronic health” OR mhealth OR m-health OR “mobile health” OR emedicine* OR e-medicine* OR “e medicine“ OR “electronic medicine” OR “e therapy“ OR “e therapies” OR etherap* OR e-therap* OR teletherap* OR “digital health” OR web-based OR “remote consultation” OR econsult* OR e-consult* OR teleconsult* OR videoconferenc* OR video-conferenc* OR teleconferenc* OR telehome OR telemanag* OR telenurs* OR telereha* OR tele-reha* OR telemonitor* OR tele-monitor* OR “home monitoring“ OR home-monitor* OR e-mail OR “electronic mail” OR “short message” OR “short messaging” OR “electronic messaging” OR SMS OR app OR “mobile application” OR “mobile applications” OR phone OR “social network“ OR e-learning OR „electronic learning”	214127
AND		
Outcome	“theory of planned behavior” OR “theory of planned behaviour” OR TPB OR “Decomposed Theory of Planned Behaviour“ OR “Theory of Interpersonal Behaviour” OR TIB OR “theory of reasoned action” OR “transtheoretical model of behavior change” OR “information-motivation-behavioral skills model” OR “social cognitive theory” OR “health belief model” OR HBM OR “technology acceptance model” OR TAM OR “unified theory of acceptance and use of technology” OR UTAUT* OR “information system success model” OR “health action process approach” OR HAPA OR “normalization process theory” OR “Theory of Diffusion of Innovations” OR “Diffusion of Innovation Theory“ OR "diffusion of innovations" OR theory OR theories OR framework* OR predict* OR determin*	4758893
	AND	
	„Patient Acceptance of Health Care“ [Mesh] OR acceptance OR accept* OR approve* OR assent OR adopt* OR “sustained use” OR sustain* OR diffus* OR "Patient Satisfaction"[Mesh] OR use OR usefulness OR usability OR satisfaction OR "attitude of health personnel" [Mesh]	2157728
NOT		
Addresses OR Autobiography OR Bibliography OR Biography OR Case Report OR Clinical Conference OR Collected Works OR Congresses OR Consensus Development Conference OR Consensus Development Conference, NIH OR Dataset OR Dictionary OR Directory OR Duplicate Publication OR Editorial OR Expression of Concern OR Festschrift OR Goverment Publications OR Guideline OR Historical Article OR Interactive Tutorial OR Interview OR Introductory Journal Article OR Lectures OR Legal Cases OR Legislation OR Letter OR Meta-Analysis OR News OR Newspaper Article OR Overall OR Patient Education Handout OR Periodical Index OR Personal Narratives OR Portraits OR Practice Guideline OR Publication Components OR Publication Formats OR Publication Type Category OR Research Support,		

American Recovery and Reinvestment Act OR Research Support, N.I.H., Extramural OR Research Support, N.I.H., Intramural OR Research Support, Non-U.S. Gov't Research Support, U.S. Gov't, Non-P.H.S. OR Research Support, U.S. Gov't, P.H.S. OR Review OR Scientific Integrity Review OR Study Characteristics OR Support of Research OR Twin Study OR Validation Studies OR Video-Audio Media OR Webcasts

Search String PsycINFO

Population	MAINSUBJECT.EXACT(“Health Personnel”) OR MAINSUBJECT.EXACT(“Patients”) OR patient OR patients OR relative* OR family OR families OR peer* OR “peer group” OR “peer groups”	1.373.333
AND		
Intervention	MAINSUBJECT.EXACT(“Telemedicine”) OR telehealth OR Telematic* OR ehealth OR e-health OR E-Health OR “electronic health” OR mhealth OR m-health OR “mobile health” OR emedicine* OR e-medicine* OR “e medicine“ OR “electronic medicine” OR “e therapy“ OR “e therapies” OR etherap* OR e-therap* OR teletherap* OR “digital health” OR web-based OR “remote consultation” OR econsult* OR e-consult* OR teleconsult* OR videoconferenc* OR video-conferenc* OR teleconferenc* OR telehome OR telemanag* OR telenurs* OR telereha* OR tele-reha* OR telemonitor* OR tele-monitor* OR “home monitoring“ OR home-monitor* OR e-mail OR “electronic mail” OR “short message” OR “short messaging” OR “electronic messaging” OR SMS OR app OR “mobile application” OR “mobile applications” OR phone OR “social network“ OR e-learning OR “electronic learning”	60.385
AND		
Outcome	“theory of planned behavior” OR “theory of planned behaviour” OR TPB OR “Decomposed Theory of Planned Behaviour“ OR “Theory of Interpersonal Behaviour” OR TIB OR “theory of reasoned action” OR “transtheoretical model of behavior change” OR “information-motivation-behavioral skills model” OR “social cognitive theory” OR “health belief model” OR HBM OR “technology acceptance model” OR TAM OR “unified theory of acceptance and use of technology” OR UTAUT* OR “information system success model” OR “health action process approach” OR HAPA OR “normalization process theory” OR “Theory of Diffusion of Innovations” OR “Diffusion of Innovation Theory” OR “diffusion of innovations” OR framework* OR predict* OR determin*	934.420
	AND	
	(MAINSUBJECT.EXACT("Compliance") OR MAINSUBJECT.EXACT("Dropouts") OR MAINSUBJECT.EXACT("Client Participation") OR (acceptance OR accept* OR approve* OR assent OR adopt* OR “sustained use” OR sustain* OR diffus*) OR MAINSUBJECT.EXACT("Client Satisfaction") OR use OR usefulness OR usability OR satisfaction OR MAINSUBJECT.EXACT(“Health Personnel Attitudes”))	1.115.275

Selected entry type: Journal, Journal Article, Peer Reviewed Journal
