# Supplementary material for: Theories Predicting End-User Acceptance of Telemedicine Use: Systematic Review
Source: J Med Internet Res. 2019 May 21;21(5):e13117. doi: 10.2196/13117 (PMC6547771; doi:10.2196/13117)
Supplement: Multimedia Appendix 2 [file jmir_v21i5e13117_app2.pdf]

	Reference	Reason for exclusion
1	Adenuga, K. I., Iahad, N. A., & Miskon, S. (2017). Towards reinforcing telemedicine adoption amongst clinicians in Nigeria. International Journal of Medical Informatics, 104, 84–96. https://doi.org/10.1016/j.ijmedinf.2017.05.008	Intervention
2	Al-Qirim, N. A. Y. (2003). Teledermatology: The case of adoption and diffusion of telemedicine health Waikato in New Zealand. Telemedicine Journal and E-Health : the Official Journal of the American Telemedicine Association, 9(2), 167–177. https://doi.org/10.1089/153056203766437507	Design
3	Andrews, L., Cacho-Elizondo, S., Drennan, J., & Tossan, V. (2013). Consumer acceptance of an SMS-assisted smoking cessation intervention: A multicountry study. Health Marketing Quarterly, 30(1), 47–62. https://doi.org/10.1080/07359683.2013.758015	Intervention
4	Angelis, G. de, Davies, B., King, J., Wells, G. A., & Brosseau, L. (2017). The use of social media by arthritis health professionals to disseminate a self-management program to patients: A feasibility study. Digital Health, 3, 2055207617700520. https://doi.org/10.1177/2055207617700520	Outcome
5	Aria, R., & Archer, N. (2018). Using an educational video vs. in-person education to measure patient perceptions of an online self-management support system for chronic illness. Computers in Human Behavior, 84, 162–170. https://doi.org/10.1016/j.chb.2018.01.041	Intervention
6	Baumeister, H., Seifferth, H., Lin, J., Nowoczin, L., Lüking, M., & Ebert, D. (2015). Impact of an Acceptance Facilitating Intervention on Patients' Acceptance of Internet-based Pain Interventions: A Randomized Controlled Trial. The Clinical Journal of Pain, 31(6), 528–535. https://doi.org/10.1097/AJP.0000000000000118	Intervention
7	Beil, J., Cihlar, V., & Kruse, A. (2015). Bereitschaft zur Akzeptanz einer internetbasierten Mobilitätsplattform bei verschiedenen Alterskohorten. Empirische Befunde des Projekts S-Mobil 100 [Willingness to accept an Internet-based mobility platform in different age cohorts. Empiric results of the project S-Mobil 100]. Zeitschrift Fur Gerontologie Und Geriatrie, 48(2), 142–149. https://doi.org/10.1007/s00391-013-0546-0	Intervention
8	Bhandari, G., & Snowdon, A. (2012). Design of a patient-centric, service-oriented health care navigation system for a local health integration network. Behaviour & Information Technology, 31(3), 275–285. https://doi.org/10.1080/0144929X.2011.563798	Intervention
9	Bhor, M., & Mason, H. L. (2006). Development and validation of a scale to assess attitudes of health care administrators toward the use of e-mail communication between patients and physicians. Research in Social & Administrative Pharmacy : RSAP, 2(4), 512–532. https://doi.org/10.1016/j.sapharm.2006.02.005	Design
10	Bischoff, C., Schmädeke, S., Fuchsloch, L. (2014). Akzeptanz Smartphone-gestützter Rehabilitationsnachsorge bei depressiven Patienten. Verhaltenstherapie und Verhaltensmedizin, 35(4), 316-333 https://www.wiso-net.de/document/VUV__BB1CB26D718E8813CD90E8F8880428E0	Outcome
11	Blomkvist, J., Aberg, J., & Holmlid, S. (2014). Formative Evaluation of IT-based Services: A Case Study of a Meal Planning Service. Interacting with Computers, 26(6), 540–556. https://doi.org/10.1093/iwc/iwt052	Intervention

12	Boessen, A. B., Vermeulen, J., & Witte, L. P. de. (2017). Acceptance and usability of a home-based monitoring tool of health indicators in children of people with dementia: A Proof of Principle (POP) study. Patient Preference and Adherence, 11, 1317–1324. https://doi.org/10.2147/PPA.S135022	Outcome
13	Borges, U., & Kubiak, T. (2016). Continuous Glucose Monitoring in Type 1 Diabetes. Journal of Diabetes Science and Technology, 10(3), 633–639. https://doi.org/10.1177/1932296816634736	Intervention
14	Brooks, E., Manson, S. M., Bair, B., Dailey, N., & Shore, J. H. (2012). The diffusion of telehealth in rural American Indian communities: A retrospective survey of key stakeholders. Telemedicine Journal and E-Health : the Official Journal of the American Telemedicine Association, 18(1), 60–66. https://doi.org/10.1089/tmj.2011.0076	Design
15	Choi, J., Baker, E., Nalawade, S., Peacock, A., Lee, H., & Choi, W. J. (2018). A Framework Facilitates Development of a Mobile App. Studies in Health Technology and Informatics, 250, 97–100.	Outcome
16	Cimperman, M., Brenčič, M. M., Trkman, P., & Stanonik, M. d. L. (2013). Older adults' perceptions of home telehealth services. Telemedicine Journal and E-Health : the Official Journal of the American Telemedicine Association, 19(10), 786–790. https://doi.org/10.1089/tmj.2012.0272	Design
17	Côté, J., Cossette, S., Ramirez-Garcia, P., Rouleau, G., Auger, P., Boudreau, F., & Gagnon, M.-P. (2017). Improving Health and Reducing Comorbidity Associated with HIV: The Development of TAVIE en santé, a Web-Based Tailored Intervention to Support the Adoption of Health Promoting Behaviors among People Living with HIV. BioMed Research International, 2017, 4092304. https://doi.org/10.1155/2017/4092304	Outcome
18	Cranen, K., Veld, R. H. I.'t., Ijzerman, M., & Vollenbroek-Hutten, M. (2011). Change of patients' perceptions of telemedicine after brief use. Telemedicine Journal and E-Health : the Official Journal of the American Telemedicine Association, 17(7), 530–535. https://doi.org/10.1089/tmj.2010.0208	Outcome
19	Deng, Z. (2013). Understanding public users' adoption of mobile health service. International Journal of Mobile Communications, 11(4), 351. https://doi.org/10.1504/IJMC.2013.055748	Paper not available
20	DiIorio, C., Reisinger, E. L., Yeager, K. A., & McCarty, F. (2009). A telephone-based self-management program for people with epilepsy. Epilepsy & Behavior : E&B, 14(1), 232–236. https://doi.org/10.1016/j.yebeh.2008.10.016	Outcome
21	Downs, D. S., Dinallo, J. M., Birch, L. L., Paul, I. M., & Ulbrecht, J. S. (2017). Randomized Face-to-Face vs. Home Exercise Interventions in Pregnant Women with Gestational Diabetes. Psychology of Sport and Exercise, 30, 73–81. https://doi.org/10.1016/j.psychsport.2017.02.003	Intervention
22	Ehrler, F., Ducloux, P., Wu, D. T. Y., Lovis, C., & Blondon, K. (2018). Acceptance of a Mobile Application Supporting Nurses Workflow at Patient Bedside: Results from a Pilot Study. Studies in Health Technology and Informatics, 247, 506–510.	Intervention
23	Emani, S., Healey, M., Ting, D. Y., Lipsitz, S. R., Ramelson, H., Suric, V., & Bates, D. W. (2016). Awareness and Use of the After-Visit Summary Through a Patient Portal: Evaluation of Patient Characteristics and an Application of the Theory of Planned Behavior. Journal of Medical Internet Research, 18(4), e77. https://doi.org/10.2196/jmir.5207	Intervention

24	FitzGerald, L. Z., Rorie, A., & Salem, B. E. (2015). Improving secondary prevention screening in clinical encounters using mhealth among prelicensure master's entry clinical nursing students. Worldviews on Evidence-Based Nursing, 12(2), 79–87. https://doi.org/10.1111/wvn.12081	Intervention
25	Forquer, H. A., Christensen, J. L., & Tan, A. S. L. (2014). Predicting continuance-findings from a longitudinal study of older adults using an eHealth newsletter. Health Communication, 29(9), 937–946. https://doi.org/10.1080/10410236.2013.833580	Intervention
26	Gallos, P., Kaitelidou, D., Velonakis, E., & Mantas, J. (2014). A "Smart" m-health Application for Travelers: The Public's Opinion. Studies in Health Technology and Informatics, 202, 245–248.	Paper not available
27	Gallos, P., & Mantas, J. (2015). The "SMART Travel Health" Mobile Application Assessment. Studies in Health Technology and Informatics, 213, 227–229.	Paper not available
28	Gatwood, J., Balkrishnan, R., Erickson, S. R., An, L. C., Piette, J. D., & Farris, K. B. (2014). Addressing medication nonadherence by mobile phone: Development and delivery of tailored messages. Research in Social & Administrative Pharmacy : RSAP, 10(6), 809–823. https://doi.org/10.1016/j.sapharm.2014.01.002	Outcome
29	Georgsson, M., & Staggers, N. (2017). Patients' Perceptions and Experiences of a mHealth Diabetes Self-management System. Computers, Informatics, Nursing : CIN, 35(3), 122–130. https://doi.org/10.1097/CIN.0000000000000296	Paper not available
30	Graaf, M. de, Totté, J. E., van Os-Medendorp, H., van Renselaar, W., Breugem, C. C., & Pasmans, S. G. (2014). Treatment of Infantile Hemangioma in Regional Hospitals With eHealth Support: Evaluation of Feasibility and Acceptance by Parents and Doctors. JMIR Research Protocols, 3(4), e52. https://doi.org/10.2196/resprot.3418	Outcome
31	Held, J. P., Ferrer, B., Mainetti, R., Steblin, A., Hertler, B., Moreno-Conde, A., . . . Borghese, N. A. (2018). Autonomous rehabilitation at stroke patients home for balance and gait: Safety, usability and compliance of a virtual reality system. European Journal of Physical and Rehabilitation Medicine, 54(4), 545–553. https://doi.org/10.23736/S1973-9087.17.04802-X	Outcome
32	Hoque, M. R., Bao, Y., & Sorwar, G. (2017). Investigating factors influencing the adoption of e-Health in developing countries: A patient's perspective. Informatics for Health & Social Care, 42(1), 1–17. https://doi.org/10.3109/17538157.2015.1075541	Intervention
33	Hoque, M. R. (2016). An empirical study of mHealth adoption in a developing country: The moderating effect of gender concern. BMC Medical Informatics and Decision Making, 16, 51. https://doi.org/10.1186/s12911-016-0289-0	Intervention
34	Horsham, C., Loescher, L. J., Whiteman, D. C., Soyer, H. P., & Janda, M. (2016). Consumer acceptance of patient-performed mobile teledermoscopy for the early detection of melanoma. The British Journal of Dermatology, 175(6), 1301–1310. https://doi.org/10.1111/bjd.14630	Outcome
35	Hou, S.-I. (2005). Stage of adoption and impact of direct-mail communications with and without phone intervention on Chinese women's cervical smear screening behavior. Preventive Medicine, 41(3-4), 749–756. https://doi.org/10.1016/j.ypmed.2005.07.011	Intervention
36	Hsieh, P.-J. (2015). Healthcare professionals' use of health clouds: Integrating technology acceptance and status quo bias perspectives.	Intervention

	International Journal of Medical Informatics, 84(7), 512–523. https://doi.org/10.1016/j.ijmedinf.2015.03.004	
37	Huang, J.-C. (2011). Exploring the acceptance of telecare among senior citizens: An application of back-propagation network. Telemedicine Journal and E-Health : the Official Journal of the American Telemedicine Association, 17(2), 111–117. https://doi.org/10.1089/tmj.2010.0118	Outcome
38	Irvine, A. B., Russell, H., Manocchia, M., Mino, D. E., Cox Glassen, T., Morgan, R., . . . Ary, D. V. (2015). Mobile-Web app to self-manage low back pain: Randomized controlled trial. Journal of Medical Internet Research, 17(1), e1. https://doi.org/10.2196/jmir.3130	Outcome
39	Jeon, E., & Park, H.-A. (2015). Factors affecting acceptance of smartphone application for management of obesity. Healthcare Informatics Research, 21(2), 74–82. https://doi.org/10.4258/hir.2015.21.2.74	Intervention
40	Ji, M., Wu, Y., Chang, P., Yang, X., Yang, F., & Xu, S. (2015). Development and Usability Evaluation of the Mobile Delirium Assessment App Based on Confusion Assessment Method for Intensive Care Unit (CAM-ICU). Studies in Health Technology and Informatics, 216, 899.	Outcome
41	Kalkhoran, S., Appelle, N. A., Napoles, A. M., Munoz, R. F., Lum, P. J., Alvarado, N., . . . Satterfield, J. M. (2016). Beyond the Ask and Advise: Implementation of a Computer Tablet Intervention to Enhance Provider Adherence to the 5As for Smoking Cessation. Journal of Substance Abuse Treatment, 60, 91–100. https://doi.org/10.1016/j.jsat.2015.05.009	Design
42	Koivumäki, T., Pekkarinen, S., Lappi, M., Väisänen, J., Juntunen, J., & Pikkarainen, M. (2017). Consumer Adoption of Future MyData-Based Preventive eHealth Services: An Acceptance Model and Survey Study. Journal of Medical Internet Research, 19(12), e429. https://doi.org/10.2196/jmir.7821	Intervention
43	Kothe, E. J., & Mullan, B. A. (2014). A randomised controlled trial of a theory of planned behaviour to increase fruit and vegetable consumption. Fresh Facts. Appetite, 78, 68–75. https://doi.org/10.1016/j.appet.2014.03.006	Intervention
44	Kowitlawakul, Y. (2011). The technology acceptance model: Predicting nurses' intention to use telemedicine technology (eICU). Computers, Informatics, Nursing : CIN, 29(7), 411–418. https://doi.org/10.1097/NCN.0b013e3181f9dd4a	Paper not available
45	Kuo, K.-M., Talley, P. C., Lee, C.-M., & Yen, Y.-C. (2015). The influence of telemedicine experience on physicians' perceptions regarding adoption. Telemedicine Journal and E-Health : the Official Journal of the American Telemedicine Association, 21(5), 388–394. https://doi.org/10.1089/tmj.2014.0091	Intervention
46	Lee, M. K., Park, H.-A., Yun, Y. H., & Chang, Y. J. (2013). Development and formative evaluation of a web-based self-management exercise and diet intervention program with tailored motivation and action planning for cancer survivors. JMIR Research Protocols, 2(1), e11. https://doi.org/10.2196/resprot.2331	Intervention
47	Lee, E., & Han, S. (2015). Determinants of adoption of mobile health services. Online Information Review, 39(4), 556–573. https://doi.org/10.1108/OIR-01-2015-0007	Outcome
48	L'Esperance, S. T., & Perry, D. J. (2016). Assessing advantages and barriers to telemedicine adoption in the practice setting: A	Design

	MyCareTeam(TM) exemplar. Journal of the American Association of Nurse Practitioners, 28(6), 311–319. https://doi.org/10.1002/2327-6924.12280	
49	Lin, T. T. C., & Bautista, J. R. (2017). Understanding the Relationships between mHealth Apps' Characteristics, Trialability, and mHealth Literacy. Journal of Health Communication, 22(4), 346–354. https://doi.org/10.1080/10810730.2017.1296508	Population
50	Liu, L., Miguel Cruz, A., Rios Rincon, A., Buttar, V., Ranson, Q., & Goertzen, D. (2015). What factors determine therapists' acceptance of new technologies for rehabilitation – a study using the Unified Theory of Acceptance and Use of Technology (UTAUT). Disability and Rehabilitation, 37(5), 447–455. https://doi.org/10.3109/09638288.2014.923529	Intervention
51	Mason, D., Gilbert, H., & Sutton, S. (2012). Effectiveness of web-based tailored smoking cessation advice reports (iQuit): A randomized trial. Addiction (Abingdon, England), 107(12), 2183–2190. https://doi.org/10.1111/j.1360-0443.2012.03972.x	Intervention
52	Miller, K. E., Kuhn, E., Owen, J. E., Taylor, K., Yu, J. S., Weiss, B. J., . . . Trockel, M. (2017). Clinician Perceptions Related to the Use of the CBT-I Coach Mobile App. Behavioral Sleep Medicine, 1–11. https://doi.org/10.1080/15402002.2017.1403326	Paper not available
53	Monthuy-Blanc, J., Bouchard, S., Maïano, C., & Séguin, M. (2013). Factors influencing mental health providers' intention to use telepsychotherapy in First Nations communities. Transcultural Psychiatry, 50(2), 323–343. https://doi.org/10.1177/1363461513487665	Population
54	Pande, T., Saravu, K., Temesgen, Z., Seyoum, A., Rai, S., Rao, R., . . . Gagnon, M.-P. (2017). Evaluating clinicians' user experience and acceptability of LearnTB, a smartphone application for tuberculosis in India. MHealth, 3, 30. https://doi.org/10.21037/mhealth.2017.07.01	Intervention
55	Parra, C., Jódar-Sánchez, F., Jiménez-Hernández, M. D., Vigil, E., Palomino-García, A., Moniche-Álvarez, F., . . . Leal, S. (2012). Development, Implementation, and Evaluation of a Telemedicine Service for the Treatment of Acute Stroke Patients: TeleStroke. Interactive Journal of Medical Research, 1(2), e15. https://doi.org/10.2196/ijmr.2163	Intervention
56	Petit-Dit-Dariel, O., Wharrad, H., & Windle, R. (2014). Using Bourdieu's theory of practice to understand ICT use amongst nurse educators. Nurse Education Today, 34(11), 1368–1374. https://doi.org/10.1016/j.nedt.2014.02.005	Intervention
57	Putzer, G. J., & Park, Y. (2012). Are physicians likely to adopt emerging mobile technologies? Attitudes and innovation factors affecting smartphone use in the Southeastern United States. Perspectives in Health Information Management, 9, 1b.	Intervention
58	Quaosar, G. M. A. A., Hoque, M. R., & Bao, Y. (2018). Investigating Factors Affecting Elderly's Intention to Use m-Health Services: An Empirical Study. Telemedicine Journal and E-Health : the Official Journal of the American Telemedicine Association, 24(4), 309–314. https://doi.org/10.1089/tmj.2017.0111	Intervention
59	Ramirez, M., Wu, S., Jin, H., Ell, K., Gross-Schulman, S., Myerchin Sklaroff, L., & Guterman, J. (2016). Automated Remote Monitoring of Depression: Acceptance Among Low-Income Patients in Diabetes Disease Management. JMIR Mental Health, 3(1), e6. https://doi.org/10.2196/mental.4823	Outcome

60	Rivas Costa, C., Fernández Iglesias, M. J., Anido Rifón, L. E., Gómez Carballa, M., & Valladares Rodríguez, S. (2017). The acceptability of TV-based game platforms as an instrument to support the cognitive evaluation of senior adults at home. PeerJ, 5, e2845. https://doi.org/10.7717/peerj.2845	Intervention
61	Rosis, S. de, & Barsanti, S. (2016). Patient satisfaction, e-health and the evolution of the patient-general practitioner relationship: Evidence from an Italian survey. Health Policy (Amsterdam, Netherlands), 120(11), 1279–1292. https://doi.org/10.1016/j.healthpol.2016.09.012	Intervention
62	Stratton, D., & Loescher, L. J. (2016). The acceptance of mobile teledermoscopy by primary care nurse practitioners in the state of Arizona. Journal of the American Association of Nurse Practitioners, 28(6), 287–293. https://doi.org/10.1002/2327-6924.12313	Outcome
63	Vanhoof, J. M. M., Vandenberghe, B., Geerts, D., Philippaerts, P., Mazière, P. de, DeVito Dabbs, A., . . . Dobbels, F. (2018). Technology Experience of Solid Organ Transplant Patients and Their Overall Willingness to Use Interactive Health Technology. Journal of Nursing Scholarship : an Official Publication of Sigma Theta Tau International Honor Society of Nursing, 50(2), 151–162. https://doi.org/10.1111/jnu.12362	Intervention
64	Van Houwelingen, C. T., Ettema, R. G., Antonietti, M. G., & Kort, H. S. (2018). Understanding Older People's Readiness for Receiving Telehealth: Mixed-Method Study. Journal of Medical Internet Research, 20(4), e123. https://doi.org/10.2196/jmir.8407	Outcome
65	Walker, B. A. (2014). The Acceptance and Use of Virtual Gaming as an Intervention Strategy for Older Adults in Occupational Therapy. Games for Health Journal, 3(6), 333–340. https://doi.org/10.1089/g4h.2014.0062	Intervention
66	Werner, P. (2004). Willingness to use telemedicine for psychiatric care. Telemedicine Journal and E-Health : the Official Journal of the American Telemedicine Association, 10(3), 286–293. https://doi.org/10.1089/tmj.2004.10.286	Outcome
67	Werner, P., & Karnieli, E. (2003). A model of the willingness to use telemedicine for routine and specialized care. Journal of Telemedicine and Telecare, 9(5), 264–272. https://doi.org/10.1258/135763303769211274	Outcome
68	Werner, P., & Korczyn, A. D. (2012). Willingness to use computerized systems for the diagnosis of dementia: Testing a theoretical model in an Israeli sample. Alzheimer Disease and Associated Disorders, 26(2), 171–178. https://doi.org/10.1097/WAD.0b013e318222323e	Outcome
69	Wilson, E. V., & Lankton, N. K. (2004). Modeling patients' acceptance of provider-delivered e-health. Journal of the American Medical Informatics Association : JAMIA, 11(4), 241–248. https://doi.org/10.1197/jamia.M1475	Intervention
70	Wynn, R., Bergvik, S., Pettersen, G., & Fossum, S. (2012). Clinicians' experiences with videoconferencing in psychiatry. Studies in Health Technology and Informatics, 180, 1218–1220.	Design
71	Zayyad, M. A., & Toycan, M. (2018). Factors affecting sustainable adoption of e-health technology in developing countries: An exploratory survey of Nigerian hospitals from the perspective of healthcare professionals. PeerJ, 6, e4436. https://doi.org/10.7717/peerj.4436	Intervention

72	Zhang, Y., & Koch, S. (2015). Mobile health apps in Sweden: What do physicians recommend? Studies in Health Technology and Informatics, 210, 793–797.	Intervention
73	Zhu, Z., Liu, Y., Che, X., & Chen, X. (2018). Moderating factors influencing adoption of a mobile chronic disease management system in China. Informatics for Health & Social Care, 43(1), 22–41. https://doi.org/10.1080/17538157.2016.1255631	Paper not available
