# Supplementary material for: Theories Predicting End-User Acceptance of Telemedicine Use: Systematic Review
Source: J Med Internet Res. 2019 May 21;21(5):e13117. doi: 10.2196/13117 (PMC6547771; doi:10.2196/13117)
Supplement: Multimedia Appendix 3 [file jmir_v21i5e13117_app3.pdf]

| Author (year); Journal | Study-year / country | Study population | | | Telemedicine application | Medical condition | Theoretical model / theory | Dependent variable | Components of the model or theory with significant explanatory power | Internal consistency / statistical analysis | Effect strength and significance |
|---|---|---|---|---|---|---|---|---|---|---|---|
| | | **No.** | **Type** | **Mean age; gender** | | | | | | | |
| | | | | | **Health care provider** | | | | | | |
| Asua et al. (2012); BMC Medical Informatics and Decision Making | 2010 / Spain | N= 605 n=268 nurses (131) general practitioners (122) pediatricians (15) | nurses, general practitioners, pediatricians | n.s.; Male: 59 (22.0%) Female: 209 (78.0%) | telemonitoring system | chronic diseases | TAM DOI TIB | Intention to use | PU (TAM) PEOU (TAM)<br><br>Extended TAM<br>Compatibility (DOI)<br>Facilitators (TIB) | reliability test (Cronbach alpha) / logistic regression | Original Technology Acceptance Model<br>PU: OR=5.28*** (CI= 3.14-10.01)<br>PEOU: OR=1.93* (CI= 1.11-2.37)<br><br>Final Model: Nagelkerke $R^2$ = 0.63<br><br>Extended TAM<br>PU: OR=2.65* (CI= 1.15-6.12)<br>Compatibility: OR=3.06** (CI= 1.30-7.18)<br>Facilitators: OR=4.90*** (CI= 2.38-10.09)<br><br>Final model: Nagelkerke $R^2$ = 0.72 |
| Gagnon et al. (2012); Telemedicine and e-health | n.s. / Spain | N=234; n=93 | nurses n=72 physicians n=21 | n.s.; Male: 17 (18.28 %) Female: 76 (81.72 | telemonitoring system | n.s. | TAM | Intention to use | PU (TAM)<br><br>modified model<br>Facilitators (TIB) | reliability test (Cronbach Alpha) / logistic regression | Original Technology Acceptance Model<br>PU: OR= 5.28*** (CI=2.12–13.11)<br><br>Nagelkerke $R^2$ = 0.42<br><br>modified model<br>Facilitators: OR= 4.96** (CI= |

| | | | | | | | | | | | | |
|---|---|---|---|---|---|---|---|---|---|---|---|---|
| | | | | %)<br>n=93 | | | | | | | | 1.59–15.55)<br><br>Final model: Nagelkerke $R^2$ = 0.54 |
| Hennemann et al. (2017), Journal of Health Communication | 2015 / 2016 / Germany | N=287<br>n=152 | health professionals of various professional groups in four inpatient rehabilitation facilities | 44.35; (n=144)<br>Male: 29 (19%) (n=149)<br>Female: 120 (81%) (n=149) | eHealth Interventions | n.s. | UTAUT | Acceptance (Behavioral Intention) | SI<br>PE | | reliability test (Cronbach alpha) / multiple linear regression | SI: beta = 0.37*** (CI= 0.25 - 0.61)<br>PE: beta = 0.28*** (CI= 0.12 - 0.44)<br><br>Final model: $R^2$ = 0.63 |
| James et al. (2016); Journal of Diabetes Science and Technology | 2014 / Australia | N=174<br>n=228 | members of the Australian Diabetes Educators Association | 47;<br>Male: 26 (11.4%)<br>Female: 102 (88.6%) | diabetes-related technologies | diabetes | TAM | Intention to use | PEOU<br>SN | | reliability test (Cronbach alpha, Exploratory factor analyses) / logistic regression | Independent Predictors of Diabetes Educators' Intentions to Use:<br><br><u>Apps</u><br>PEOU: OR=1.15* (CI=1.07-1.31)<br>Final model: $R^2$=0.71<br><br><u>Video conferencing</u><br>PEOU: OR=1.21*** (CI=1.08-1.35)<br>SN: OR=1.21** (CI=1.07-1.37)<br>Final model: $R^2$=0.68 |
| Kuhn et al. (2015); Professional Psychology: Research and Practice | n.s. / USA | N=1257<br>n=271 | mental health clinicians | 47.17;<br>Male: 99 (36.5)<br>Female: 172 (63.5) | PE (Prolonged Exposure) Coach Mobile App | posttraumatic stress disorder | DOI | Use | complexity | | reliability test (Cronbach alpha) / logistic regression analysis | complexity: OR = .35*** (CI=0.23- 0.55)<br><br>Final Model: Nagelkerke $R^2$=0.53 |

| | | | | | | | | | | | |
|---|---|---|---|---|---|---|---|---|---|---|---|
| Orruño et al. (2011), Journal of Telemedicine and Telecare | n.s. / Spain | N=276<br>n=171 | physicians | n.s.;<br>Male: 72 (42%)<br>Female: 99 (58%) | teledermatology | skin lesions | TAM<br>TIB<br>TRA | Intention to use | PU (TAM)<br>PEOU (TAM)<br>facilitators (TIB) | reliability test (Cronbach alpha) / logistic regression | Original Technology Acceptance Model<br>PU -> intention: OR= 8.4, *** (CI=3.4–21.0)<br>PEOU -> intention: OR= 7.4, *** (CI=2.9–19.0)<br><br>Nagelkerke's $R^2$=0.71<br><br>Modified Technology Acceptance Model<br>Facilitators -> intention: OR= 9.9*** (CI=2.80–34.94)<br><br>Final Model: Nagelkerke's $R^2$=0.78 |
| Saigi-Rubió et al. (2014); Implementation Science | 2012 / Spain, Bolivia, Columbia | N=890<br>n=310<br>N (Spain) =356<br>n (Spain) =113<br>N (Bolivia) =350<br>n (Bolivia) =279<br>N (Columbia)=184<br>n (Columbia)=118 | physicians | n.s.;<br>Male (Spain): 67.3%<br>Female (Spain): 32.7%<br>Male (Bolivia): 40.5%<br>Female (Bolivia): 59.5%<br>Male (Columbia): 60.2%<br>Female (Columbia): 39.8% | telemedicine | n.s. | TAM<br>DOI<br>TRA<br>TPB<br>TR | Use | Level of ICT use (TR)<br>Optimism (TR) | reliability test (Cronbach alpha, Exploratory factor analyses) / logistic regression | Technology Readiness Index<br>Level of ICT Use (Spain): b=2.661**<br>Level of ICT Use (Columbia): b=1.212**<br>Optimism (Bolivia): b=0.484***<br><br>Final Model:<br>Nagelkerke’s $R^2$ (Spain): 0.275<br>Nagelkerke’s $R^2$ (Columbia): 0.161<br>Nagelkerke’s $R^2$ (Bolivia): 0.197 |

| | | | | | | | | | | | |
|---|---|---|---|---|---|---|---|---|---|---|---|
| Saigi-Rubió et al. (2016); International Journal of Technology Assessment in Health Care | 2012 / Spain | N=398<br>n=96 | physicians (random sample) | did not exceed 40 years;<br>Male: 26 (26.9%)<br>Female: 70 (73.1%) | telemedicine | n.s. | TAM, TPB, TRA | Intention to use | PU (cost reduction, quality of care) (TAM)<br>ATT (confidentiality and security) (TAM)<br>SN (patients, medical staff) (TRA) | n.s. / logistic regression | PU (cost reduction) -> BI: b=1.342*<br>ATT (security & confidentiality) -> BI: b=0.798*<br>SN (patients) -> BI: b=0.583**<br>SN (medical staff) -> BI: b=1.005**<br><br>moderations:<br>SN (patients)*PU (quality of care) -> BI: b=0.347**<br>SN (patients)*PU (cost reduction) -> BI: b=0.462*<br>SN (medical stuff)*PU (quality of care) -> BI: b=0.366*<br>SN (medical stuff)*PU (cost reduction) -> BI: b=0.488*<br>SN (administration)*PU (cost reduction) -> BI: b=0.571*<br><br>Final model: Nagelkerke $R^2$ = 0.481<br><br>CI n.s. |
| Spaulding et al. (2005); Journal of Telemedicine and Telecare | n.s. / USA | N=356<br>n=186 | physicians and physician's assistants | n.s.;<br>Male: 141 (76%)<br>Female: 45 (24%) | telemedicine | n.s. | DOI | number of TM referals | relative advantage (provider)<br>relative advantage (patient)<br>observability<br>trialability<br>opinion leader present | n.s. / bivariate correlation analysis (Pearson's r)<br>group differences | relative advantage (provider): r=0.42*<br>relative advantage (patient): r=0.42*<br>observability: r=0.57*<br>triability: r=0.44*<br>opinion leader present: r=0.52*<br><br>CI n.s. |

| | | | | | | | | | | | |
|---|---|---|---|---|---|---|---|---|---|---|---|
| van Houwelingen et al. (2015); Journal of Gerontological Nursing | 2012 / Netherlands | N=n.s. n=207 | nurses | 43.54; Male: 11 (5,5%) Female: 190 (94.5%) bei n=207 | home telehealth | n.s. | UTAUT | Willingness to use home telehealth Actual use of home telehealth | PU EE SI | reliability test (Cronbach alpha) / multiple linear regression | PU: beta=0.435*** EE: beta=0.28*** SI: beta=0.216* Final model: $R^2$=0.54 CI n.s. |
| Vanneste et al. (2013); BMC Medical Informatics and Decision Making | n.s. / Belgium | N=661 n=282 | nurses, physical therapists, occupational therapists, speech-language therapists, dieticians, podiatrists, social workers, physicians, psychologists, dentists, pharmacists | n.s.; Male: 66 (23.4%) Female: 216 (76.6%) | BelRAI web application | age-related disabilities | UTAUT SCT | Behavioral intention | FC (UTAUT) SE (SCT) | reliability test (Cronbach alpha, Confirmatory factor analysis) / SEM | FC: beta=0.287*** SE: beta=0.218*** Final model: $R^2$=0.308 CI n.s. |
| Zhang et al. (2010); Computers, Informatics, Nursing | n.s. / Canada | N=91 n=84 | home care nurses | n.s. | mobile information technology | wounds | TAM 2 | Adoption intention | SN IM PEOU PU | reliability test (Cronbach's Alpha, confirmatory factor analysis) / SEM | SN: beta=0.323** IM: beta=0.227** PEOU: beta=0.35** PU: beta=0.422*** $R^2$=0.375 |

| | | | | | | | | | | | |
|---|---|---|---|---|---|---|---|---|---|---|---|
| | | | | | | | | | | | CI n.s. |
| **Patients** | | | | | | | | | | | |
| Cajita et al. (2017); Journal of Cardiovascular Nursing | 2016 / USA | study sample: N=129<br><br>in-person group: N=562; n=29<br><br>online group: N=188; n=100 | older adults with HF | 71.3; Male: 95 (73.6%) Female: 34 (26.4%) | mHealth | heart failure | TAM<br>TIB | Intention to use mHealth | PEOU (TAM)<br>PU (TAM) | reliability test (Cronbach alpha) / linear regression | Block 5: change in $R^2$=0.095***<br>PEOU: beta=0.16*** (CI=0.07-0.24),<br>Block 6: change in $R^2$=0.130***<br>PU: beta=0.33*** (CI=0.24-0.41),<br><br>Final model: $R^2$=0.353 |
| de Veer et al. (2015); BMC Health Services Research | n.s. / Netherlands | N=150<br>n=1014 | sample from the Dutch Health Care Consumer Panel; stratified random sample | n.s.; Male: 518 (51.1%) Female: 496 (48.9%) | eHealth application | n.s. | UTAUT | Intention to use eHealth | PE<br>EE<br>SE | reliability test (Cronbach alpha) / linear regression | PE: Block2: beta = 0.52***, Block 3: beta = 0.24***, Block 4: beta = 0.24*** Final model: beta = .24***<br>EE: Block 3: beta = 0.42***, Block 4: beta = 0.42***, Final model: beta = 0.35***<br>SE: Final model= .01**<br><br>Final model: $R^2$=0.41<br><br>CI n.s. |
| Dockweiler et al. (2017); Gesundheitswesen | 2015 / 2016 / Germany | N=379<br>n=186 | persons with current or past depressive disorder | n.s.; Male: 62 (33.3%) Female: 124 (66.7%) | e-Mental-Health (online based therapy offers) | mild to moderate depressive disorders | UTAUT | Intention to use | PE<br>EE | reliability test (Cronbach alpha) / logistic regression | PE (5 significant variables) average effect: OR=11,325* (CI= 2,666 - 49,015)<br><br>EE (2 significant variables) average effect: OR=0,121* (CI= 0,022 - 0,685)<br><br>Final model: $R^2$= 0.765 |

| | | | | | | | | | | | |
|---|---|---|---|---|---|---|---|---|---|---|---|
| Dou et al. (2017); JMIR Mhealth Uhealth | 2016 / China | N=279<br>n=152 | outpatients | n.s.;<br>Male: 106 (69.7%)<br>Female: 46 (30.3%) | smartphone health technology (smartphone health app Blood Pressure Assistant; Web-based physician portal) | hypertension | TAM<br>TAM 2<br>Dual-Factor model<br>HBM | Intention to use, actual use | PU (TAM)<br>PHT (HBM)<br>resistance to change (Dual-Factor Model) | reliability test (Explorative factor analysis) / path modeling | PU -> intention to use: beta=.616**<br>PHT -> intention to use: beta=.305**<br>resistance to change -> intention to use: beta=−.149*<br><br>Final model: $R^2$=0.412<br><br>CI n.s. |
| Hennemann et al. (2016); Journal of Medical Internet Research | 2015 / 2016 / Germany | N=977<br>n=374 | inpatients | 45.49;<br>Male: 198 (58.6%)<br>Female: 140 (41.4%) bei n=338 | web-based aftercare | occupational stress | UTAUT | Acceptance (Behavioral Intention) | SI<br>PE<br>EE | reliability test (Cronbach Alpha) / multiple linear regression | SI: beta=.39** (CI=0.3 - 0.54)<br>PE: beta=.31*** (CI=0.19 - 0.43)<br>EE: beta=.22*** (CI=0.09 - 0.31)<br><br>Final model: $R^2$=0.78 |
| Hossain et al. (2018); Telemedicine and e-Health | 2016 / Bangladesh | N=n.s.<br>n=292 | rural patients (random sample) | n.s.;<br>Male: 205 (70%)<br>Female: 87 (30%) | eHealth (teleconsultancy) | n.s. | UTAUT<br>TAM | eHealth Acceptance | social reference (means SI) (UTAUT)<br>ATT (TAM)<br>FC (UTAUT) | reliability test (Cronbach alpha, Explorative factor analysis) / logistic regression | SR: OR = 9.73** (CI=4.16 - 22.78)<br>ATT: OR= 4.56** (CI= 2.71 - 7.66)<br>FC: OR = 3.92* (CI=1.29 - 11.95)<br><br>Final model: $R^2$ = 0.55 |
| Huygens et al. (2015); Interactive Journal of Medical Research | 2013 / Netherlands | N=1500<br>n=546 | members of the Dutch Health Care | 53.14;<br>Male: 238 (43.6%) (n=238)<br>Female: | internet service for communication with | n.s. | UTAUT | Intention to use | EE<br>PE<br>FC<br>SI<br>ATT | n.s. / univariate logistic regression | service to ask questions by Internet via email or a website:<br>EE: OR=5.46 (CI=3.27-9.13)<br>PE: OR=5.47 (CI=3.44-8.70)<br>ATT: OR=5.85 (CI=3.63- |

| | | | | | | | | | | | |
|---|---|---|---|---|---|---|---|---|---|---|---|
| | | | Consumer Panel | 308 (66.8%) (n=238) | general practice | | | | | | 9.43)<br>FC: OR=7.91 (CI=4.53-13.82)<br>SI: OR=4.34 (CI=2.46-7.68)<br><br>not levels of significance reported |
| Lin & Yang (2009); Telemedicine and e-Health | n.s. / Taiwan | N=500<br>n=229 | patients | n.s.;<br>Male: 145 (63.3%)<br>Female: 84 (36.7%) | eHealth (Asthma Care Mobile Service Model) | asthma | TAM | Behavioral Intention to use | PU<br>ATT<br>SN<br>PEOUxATT<br>PUxATT<br>SNxATT | reliability test (Cronbach alpha, confirmatory factor analysis) / SEM | Direct effects:<br>ATT -> BI (lambda = 0.76**)<br>SN -> BI (lambda = 0.16*)<br><br>Total effects:<br>ATT -> BI (lambda = 0.76**)<br>PU -> BI (lambda = 0.62**)<br>SN -> BI (lambda = 0.42**)<br>PEOU -> BI (lambda = 0.3**)<br><br>Final Model: $R^2$=0.8<br><br>CI n.s. |
| Peeters et al. (2012); Journal of Clinical Nursing | 2007 / Netherlands | N=468<br>n=254 | older chronically ill clients | 77.8; (n=248)<br>Male: 81 (32.1%)<br>Female: 171 (67.9%)<br>n=252 | home telecare | chronic diseases | DOI | Adoption | relative advantage<br>compatibility<br>complexity<br>observability | reliability test (Cronbach alpha) / multiple linear regression | relative advantage: beta = 0.17*<br>compatibility: beta = 0.2**<br>complexity: beta = 0.19**<br>observability: beta = 0.34***<br><br>Final model: $R^2$=0.61<br><br>CI n.s. |
| Rho et al. (2015); Cluster Computing | 2011 / Korea | N=200<br>n=116 | outpatients | n.s.;<br>Male: 62 (53.4%)<br>Female: 54 (46.6%) | telemedicine service | diabetes | UTAUT | Behavioral Intention to use | PE<br>EE<br>SI<br>FC -> EE<br>FC -> PE | reliability test (Cronbach alpha, confirmatory factor analysis) / SEM | PE: beta=0.345**<br>EE: beta=0.227*<br>SI: beta=0.246*<br>FC*PE -> BI: beta=0.176**<br>FC*EE -> BI : beta=0,153**<br><br>Final model: $R^2$=0.44<br><br>CI n.s. |

| | | | | | | | | | | | |
|---|---|---|---|---|---|---|---|---|---|---|---|
| Zhang et al. (2017); Informatics for Health and Social Care | n.s. / China | N=n.s. n=650 | patients | n.s.; Male: 368 (56.6%) Female: 232 (35.7%) | mobile health service | chronic diseases | TAM SCT PMT | Adoption intention | PU (TAM)<br>PEOU (TAM)*PU<br>SE (Protection Motivation Theory)*PEOU*PU* AI<br>RE*PEOU*PU*AI<br>RE (Protection Motivation Theory)*PEOU*PU* AI | reliability test (confirmatory factor analysis) / SEM | <u>direct effect:</u><br>PU: beta=0.3***<br><u>moderator:</u><br>PU x SE: beta=0.145**<br>PU x RE: beta=0.359 ***<br><br>Final model: $R^2$=0.501<br><br>CI n.s. |
| **Social environment** | | | | | | | | | | | |
| Jen & Hung (2010); Telemedicine and e-Health | n.s. / Taiwan | N=192 n=100 | family member | n.s.; Male: 59 (59%) Female: 41 (41%) | mobile health care service | chronic diseases | TPB, TAM | Behavioral Intention of adopting MHS | ATT<br>PU<br>PEOU | reliability test (Exploratory factor analysis) / SEM | BI of adopting MHS is explained directly by ATT<br><br>ATT -> BI (beta=0.547**)<br><br>Final model: $R^2$=0.641 of the variance in BI<br><br>CI n.s. |
