# Supplementary material for: Theories Predicting End-User Acceptance of Telemedicine Use: Systematic Review
Source: J Med Internet Res. 2019 May 21;21(5):e13117. doi: 10.2196/13117 (PMC6547771; doi:10.2196/13117)
Supplement: Multimedia Appendix 4 [file jmir_v21i5e13117_app4.pdf]

Appendix 3 list of combined models and theories

Theory	Frequency of use	Variance explained
TAM + TIB + TRA	1	0.780
TAM + DOI + TIB	1	0.720
TPB + TAM	1	0.641
UTAUT + TAM	1	0.550
TAM + TIB	1	0.540
TAM +SCT + PMT	1	0.501
TAM + TPB + TRA	1	0.481
TAM + HBM + Dual factor model	1	0.412
TAM + DOI + TRA + TPB + TR	1	Spain: 0.275<br>Columbia: 0.161<br>Bolivia: 0.197
UTAUT + SCT	1	0.308

TAM=Technology Acceptance Model; DOI=Diffusion of Innovations; TIB= Theory of Interpersonal Behavior; HBM=Health Belief Model; UTAUT=Unified Theory of Acceptance and Use of Technology; TPB=Theory of Planned Behavior; TRA=Theory of Reasoned Action; SCT=Social Cognitive Theory; PMT=Protection Motivation Theory; TR=Technology Readiness Index
