# Supplementary material for: Theories Predicting End-User Acceptance of Telemedicine Use: Systematic Review
Source: J Med Internet Res. 2019 May 21;21(5):e13117. doi: 10.2196/13117 (PMC6547771; doi:10.2196/13117)
Supplement: Multimedia Appendix 5 [file jmir_v21i5e13117_app5.pdf]

Study	Introduction	Methods					
	1. Were the aims/objectives of the study clear?	2. Was the study design appropriate for the stated aim(s)?	3. Was the sample size justified?	4. Was the target/reference population clearly defined? (Is it clear who the research was about?)	5. Was the sample frame taken from an appropriate population base so that it closely represented the target/reference population under investigation?	6. Was the selection process likely to select subjects/participants that were representive of the target/reference population under investigation?	7. Were measures undertaken to address and categorize non-responders?
Asua et al. (2012)	1	1	1	1	1	0	1
Cajita et al. (2017)	1	1	1	1	1	1	1
de Veer et al. (2015)	1	1	1	1	1	1	0
Dockweiler et al. (2017)	1	1	1	1	1	1	0
Dou et al. (2017)	1	1	1	1	1	0	0
Gagnon et al. (2012)	1	1	1	1	1	0	0
Hennemann et al. (2016)	1	1	1	1	1	1	0
Hennemann et al. (2017)	1	1	1	1	1	1	0
Hossain et al. (2018)	1	1	1	1	1	1	0
Huygens et al. (2015)	1	1	1	1	1	1	0

James et al. (2016)	1	1	1	1	1	1	1
Jen & Hung (2010)	1	1	1	1	0	1	0
Kuhn & Reger (2015)	1	1	1	1	1	1	1
Lin & Yang (2009)	1	1	1	1	1	0	0
Orruño et al. (2011)	1	1	1	1	1	0	0
Peeters et al. (2012)	1	1	1	1	1	1	0
Rho et al. (2015)	1	1	1	1	1	1	0
Saigí-Rubió et al. (2014)	1	1	1	1	1	1	0
Saigí-Rubió & Jiménez-Zarco (2016)	1	1	1	0	1	1	0
Spaulding et al. (2005)	1	1	1	1	1	1	0
van Houwelingen et al. (2015)	1	1	1	1	1	1	0
Vanneste et al. (2013)	1	1	1	1	1	1	1
Zhang et al. (2010)	1	1	1	1	1	1	0
Zhang et al. (2016)	1	1	1	0	1	1	0

Study					Results		
	8. Were the risk factor and outcome variables measured appropriate to the aims of the study?	9. Were the risk factor and outcome variables measured correctly using instruments/measurements that have been trialled, piloted, or published previously?	10. Is it clear what was used to determine statistical significance and/or precision estimates? (eg, p values, Cis)	11. Were the methods (including statistical methods) sufficiently described to enable them to be repeated?	12. Were the basic data adequately described?	13. Does the response rate raise concerns about non-response bias?*	14. If appropriate, was information about non-responders described?
Asua et al. (2012)	1	1	1	1	1	1	1
Cajita et al. (2017)	1	1	1	1	1	0	1
de Veer et al. (2015)	1	1	1	0	1	0	0
Dockweiler et al. (2017)	1	1	1	1	1	0	1
Dou et al. (2017)	1	1	1	0	1	0	0
Gagnon et al. (2012)	1	1	1	1	1	1	0
Hennemann et al. (2016)	1	0	1	1	1	1	1
Hennemann et al. (2017)	1	0	1	1	1	0	1
Hossain et al. (2018)	1	1	1	1	1	0	0
Huygens et al. (2015)	1	0	1	0	1	1	1

James et al. (2016)	1	1	1	1	1	1	0
Jen & Hung (2010)	1	1	1	0	1	1	0
Kuhn & Reger (2015)	1	1	1	1	1	1	0
Lin & Yang (2009)	1	1	1	0	1	0	0
Orruño et al. (2011)	1	1	1	1	1	0	1
Peeters et al. (2012)	1	1	1	0	1	0	0
Rho et al. (2015)	1	1	1	0	1	0	0
Saigí-Rubió et al. (2014)	1	1	0	1	1	0	0
Saigí-Rubió & Jiménez-Zarco (2016)	1	0	1	0	1	1	0
Spaulding et al. (2005)	1	0	1	0	0	0	0
van Houwelingen et al. (2015)	1	1	1	0	1	99	0
Vanneste et al. (2013)	1	1	1	0	1	0	0
Zhang et al. (2010)	1	1	1	0	0	0	0
Zhang et al. (2016)	1	1	1	0	1	99	0

Study			Discussion		Other		AXIS Score /20
	15. Were the results internally consistent?	16. Were the results presented for all the analyses described in the methods?	17. Were the authors discussions and conclusions justified by the results?	18. Were the limitations of the study discussed?	19. Were there any funding sources or conflicts of interest that may affect the authors' interpretation of the results?*	20. Was ethnical approval or consent of participants attained?	
Asua et al. (2012)	1	0	0	1	1	1	15/20
Cajita et al. (2017)	1	1	1	1	1	1	19/20
de Veer et al. (2015)	1	1	1	1	0	1	17/20
Dockweiler et al. (2017)	1	0	1	1	0	1	18/20
Dou et al. (2017)	1	1	1	1	0	1	16/20
Gagnon et al. (2012)	1	1	1	1	0	1	16/20
Hennemann et al. (2016)	1	1	1	1	0	1	17/20
Hennemann et al. (2017)	1	0	1	1	0	1	17/20
Hossain et al. (2018)	1	1	1	1	0	0	17/20
Huygens et al. (2015)	0	0	0	1	0	0	12/20

James et al. (2016)	1	1	1	1	0	1	18/20
Jen & Hung (2010)	1	1	1	1	0	0	14/20
Kuhn & Reger (2015)	1	1	1	1	99	1	17/20
Lin & Yang (2009)	1	1	1	1	0	0	15/20
Orruño et al. (2011)	1	1	1	1	1	0	16/20
Peeters et al. (2012)	1	1	1	1	1	0	15/20
Rho et al. (2015)	1	1	1	1	1	0	15/20
Saigí-Rubió et al. (2014)	1	0	1	1	0	0	15/20
Saigí-Rubió & Jiménez-Zarco (2016)	1	1	1	0	0	0	12/20
Spaulding et al. (2005)	1	1	1	0	99	1	13/20
van Houwelingen et al. (2015)	1	1	1	1	99	1	15/20
Vanneste et al. (2013)	1	1	1	1	0	1	18/20
Zhang et al. (2010)	1	1	1	1	99	0	14/20
Zhang et al. (2016)	1	1	1	1	0	1	15/20
							15,67/20

Key: 1="Yes", 0="No", 99="Don't know"
*Item is reverse scored (i.e., 0 is a positive, counts as a point)
99 counts as a 0
